# Comprehensive research into prognostic and immune signatures of transcription factor family in breast cancer

**DOI:** 10.1186/s12920-023-01521-y

**Published:** 2023-04-25

**Authors:** Qing Wu, Shiyao Zheng, Nan Lin, Xianhe Xie

**Affiliations:** 1 Department of Oncology, Molecule Oncology Research Institute, The First Affiliated Hospital of Fujian Medical University, No. 20 Chazhong Road, Fuzhou, 350005 Fujian China; 2 Department of Oncology, National Regional Medical Center, Binhai Campus of the First Affiliated Hospital, Fujian Medical University, Fuzhou, 350212 China; 3 College of Clinical Medicine for Oncology, Fujian Medical University, Fuzhou, Fujian China; 4 Fuzong Clinical Medical College of Fujian Medical University, Fuzhou, Fujian China; 5 Department of Gastrointestinal Surgery, The 900th Hospital of Joint Logistics Support Forces of Chinese PLA, Fuzhou, Fujian China; 6 Fujian Key Laboratory of Precision Medicine for Cancer, The First Affiliated Hospital of Fujian Medical University, Fuzhou, 350005 China

**Keywords:** Transcription factor family, Breast cancer, Prognostic signature, Tumor immune microenvironment, Immune checkpoints

## Abstract

**Background:**

Breast cancer (BRCA) is the most common malignancy with high morbidity and mortality in women, and transcription factor (TF) is closely related to the occurrence and development of BRCA. This study was designed to identify a prognostic gene signature based on TF family to reveal immune characteristics and prognostic survival of BRCA.

**Methods:**

In this study, RNA-sequence with corresponding clinical data were obtained from The Cancer Genome Atlas (TCGA) and GSE42568. Prognostic differentially expressed transcription factor family genes (TFDEGs) were screened to construct a risk score model, after which BRCA patients were stratified into low-risk and high-risk groups based on their corresponding risk scores. Kaplan–Meier (KM) analysis was applied to evaluate the prognostic implication of risk score model, and a nomogram model was developed and validated with the TCGA and GSE20685. Furthermore, the GSEA revealed pathological processes and signaling pathways enriched in the low-risk and high-risk groups. Finally, analyses regarding levels of immune infiltration, immune checkpoints and chemotactic factors were all completed to investigate the correlation between the risk score and tumor immune microenvironment (TIME).

**Results:**

A prognostic 9-gene signature based on TFDEGs was selected to establish a risk score model. According to KM analyses, high-risk group witnessed a significantly worse overall survival (OS) than low-risk group in both TCGA-BRCA and GSE20685. Furthermore, the nomogram model proved great possibility in predicting the OS of BRCA patients. As indicted in GSEA analysis, tumor-associated pathological processes and pathways were relatively enriched in high-risk group, and the risk score was negatively correlated with ESTIMATE score, infiltration levels of CD4+ and CD8+T cells, as well as expression levels of immune checkpoints and chemotactic factors.

**Conclusions:**

The prognostic model based on TFDEGs could distinguish as a novel biomarker for predicting prognosis of BRCA patients; in addition, it may also be utilized to identify potential benefit population from immunotherapy in different TIME and predict potential drug targets.

**Supplementary Information:**

The online version contains supplementary material available at 10.1186/s12920-023-01521-y.

## Introduction

To date, breast cancer (BRCA) has been the most common cancer among women worldwide, with high morbidity and mortality [1]. Over the last several decades, significant progress has been made in the field of BRCA treatment, including surgery, radiotherapy, chemotherapy, hormonal therapy, targeted therapy, immunotherapy and so on [2–7]. Nevertheless, the mortality for BRCA remains high due to its complicated pathogenesis, development and metastasis [8].

As is known to all, transcription factors (TFs) play an important role in promoting or inhibiting downstream genes via binding specific sequences, with significant influence on the occurrence, migration, invasion and other biological processes of tumors [9]. Meanwhile, TFs are reported to be crucial to the occurrence and development of BRCA [10, 11]. Recently, the role of tumor immune microenvironment (TIME) in BRCA and its effect on tumor progression have increasingly aroused general concern [12, 13]. It is worth noting that, TFs can also affect the prognosis of BRCA by various TIME, with specific mechanism remaining unclear [14, 15]. Therefore, it is urgently required to explore the underlying molecular mechanisms and prognostic indicators for patients with BRCA.

In some previous studies, bioinformatics analysis was completed based on prognosis model of malignant tumors in terms of lncRNA, circRNA or mRNA [16–19]. In our study, we acquired RNA-sequence with corresponding clinicopathological data of BRCA patients from The Cancer Genome Atlas (TCGA) and GSE42568, after which we conducted a comprehensive bioinformatics analysis based on levels of gene-expression, Venn analysis and univariate Cox analysis with clinical data from TCGA-BRCA. By selecting prognostic differentially expressed transcription factor family genes (TFDEGs), we managed to set up a risk score system of BRCA for validation in cohorts of TCGA and GSE20685. To further investigate the potential mechanisms and pathways thereof, the function and gene set enrichment analyses (GSEA) were completed on the basis of differentially expressed genes (DEGs) from high-risk group compared with low-risk group; meanwhile, Connectivity Map (CMap) database was used to predict potential drug targets for high-risk group. In addition, various analyses with regard to tumor-infiltrating immune cells (TIICs), immune checkpoints and chemotactic factors were adopted to clarify the correlation between the risk score and TIME.

## Materials and methods

### Data sources

RNA-sequence with corresponding clinical data of BRCA and normal samples were obtained from TCGA (https://portal.gdc.cancer.gov/) [20], consisting of 1109 BRCA patients with comprehensive profiles of gene expression and clinical characteristics, 40 of which were removed for incomplete transcriptomic and clinical information. After that, the remaining 1069 BRCA patients with complete information were included as training set for further analyses. The GSE42568 set, including 104 BRCA samples and 17 normal samples, was downloaded from Gene Expression Omnibus (GEO) (https://www.ncbi.nlm.nih.go/geo/), while TF family including 1536 genes were retrieved from the Molecular Signatures Database (MSigDB) (www.gsea-msigdb.org/gsea/msigdb/human/gene_familes.jsp). In addition, the cohort of GSE20685 (involving 327 BRCA samples) in GEO (https://www.ncbi.nlm.nih.go/geo/) was regarded as the testing set, including RNA-sequencing configuration files and clinical information.

### Selection and functional clustering analyses of TFDEGs

The DEGs between tumor and normal samples were identified using the “DESeq2” package in TCGA-BRCA and “GEO2R” in GSE42568 with thresholds of |log2 (fold-change)|values > 1 and adjusted *P* < 0.05. Meanwhile, Venn analysis was applied to select overlapping DEGs among three algorithms mentioned above, and the “ggplot2” package to generate Volcano Plot and Differential Ranking Chart of the TFDEGs. By means of “clusterProfiler” package (version 3.14.3), Gene Ontology (GO) and Kyoto Encyclopedia of Genes and Genomes (KEGG) pathway enrichment analyses (www.kegg.jp/kegg/kegg1.html) were performed for patients based on the 113 TFDEGs [21]. Moreover, a protein–protein interaction (PPI) network of 113 TFDEGs was generated via Search Tool for the Retrieval of Interacting Genes (STRING) (https://string-db.org).

### Constructing and validating risk score model

Based on clinical data of TCGA-BRCA, univariate Cox analysis of overall survival (OS) was applied to identify TFDEGs with prognostic values and then visualized by forest plot (*P* < 0.05) [22]. Least Absolute Shrinkage and Selection Operator (LASSO) regression model was conducted to reduce the overfitting high-dimensional prognostic genes [23]. Then, the screened genes were integrated into a risk signature, after which a risk score system was established based on the normalized values of gene expression and coefficients in accordance with the following formula.

$${\text{Risk}}\;{\text{score}} = \mathop \sum \limits_{i = 1}^{n} expression_{gene\_i} \times lasso\_coeffieicent_{gene\_i}$$

The risk score was calculated for each BRCA patient, after which patients were divided into low-risk and high-risk groups according to the median risk score of TCGA-BRCA. Furthermore, patients were also assigned into subgroups by clinicopathological characteristics [including age, T stage, N stage, M stage, TNM stage, Menopause status (Pre: < 6 months since last menstrual period AND no prior bilateral ovariectomy AND not on estrogen replacement; Post: prior bilateral ovariectomy OR > 12 months since last menstrual period with no prior hysterectomy), and PAM50], so as to identify the correlation between risk score and clinical features. In addition, Kaplan–Meier (KM) analysis was utilized to evaluate the relationship between risk groups and OS of different groups.

### Nomogram

To assess whether the risk score model could serve as an independent predictive indicator, univariate and multivariate Cox regression analyses were completed with clinicopathological indicators, including the age, T stage, N stage, M stage, status of estrogen receptor (ER), progesterone receptor (PR), human epidermal growth factor receptor 2 (HER2), as well as menopause. All the independent prognostic clinicopathological parameters and risk scores were summarized to establish a nomogram for predicting the OS rates of 2-, 3- and 5-year. Furthermore, calibration, time-dependent receiver operating characteristic (ROC) curves and concordance index (C-index) were applied to assess the discriminatory ability of the nomogram.

### Analyses of 9 TFDEGs in risk score model

We performed differential expression analysis of identified risk genes in TCGA-BRCA, among which GO and KEGG pathway enrichment analyses with *P* values < 0.05 were regarded as statistically significant. Furthermore, Human Protein Atlas (HPA) database (http://www.proteinatlas.org/) was applied to evaluate the protein expression of the risk genes.

DNA methylation plays a key role in prognostic assessment and potential biomarker in cancer development [24]. MethSurv (https://biit.cs.ut.ee/methsurv/) was adopted in this study to determine the expression and prognostic patterns of single CpG methylation of the TFDEGs in BRCA [25]. In this analysis, DNA methylation values were represented by beta values ranging from 0 to 1.

### Analysis of DEGs from high-risk group

DEGs from high-risk group compared with low-risk group in TCGA-BRCA were screened by R packages mentioned above, after which GO and KEGG pathway enrichment analyses with *P* < 0.05 were considered statistically significant. In addition, GSEA (http://software.broadinstitute.org/gsea/index.jsp) (version 3.14.3) [26] was applied to identify hallmarks of high-risk group, comparing with low-risk group, and get visualized by ridge map. Finally, a signature of DEGs from high-risk group compared with low-risk group was used to predict potential drug targets for BRCA patients in high-risk group through CMap database (https://clue.io) [27].

### The correlation between prognostic model and TIME

As is well-known to all, the tumor infiltrates immune cells resulting in great threaten to patients' survival. The immune infiltration scores of TCGA-BRCA samples were computed by ESTIMATE [28], and the infiltration levels of 24 types of immune cells in BRCA samples were calculated through Immune Cell Abundance Identifier (ImmuCellAI) (http://bioinfo.life.hust.edu.cn/ImmuCellAI) [29]. In addition, we also validated the correlation between identified genes (TFDEGs) and immune cells by means of the "TIMER" (http://timer.cistrome.org/) analysis tool [30]. To further predict the TIME, the correlations of prognostic model with the expression of immune checkpoints and chemotactic factors were analyzed based on TCGA-BRCA.

### Statistical analysis

Statistical analyses in this study were performed via R software (version 3.6.3) (R code, data input, and output of this study were provided in Additional file 1: R code and data), with log-rank test for the KM analysis. Furthermore, T test or Wilcoxon test was adopted to evaluate the differences in the risk score among various clinical characteristic subtypes, as well as those differences in the level of immune infiltration, immune checkpoints and chemotactic factors between the low-risk and high-risk groups. *P < 0.05, **P < 0.01, ***P < 0.001.

## Results

**Fig. 1:**
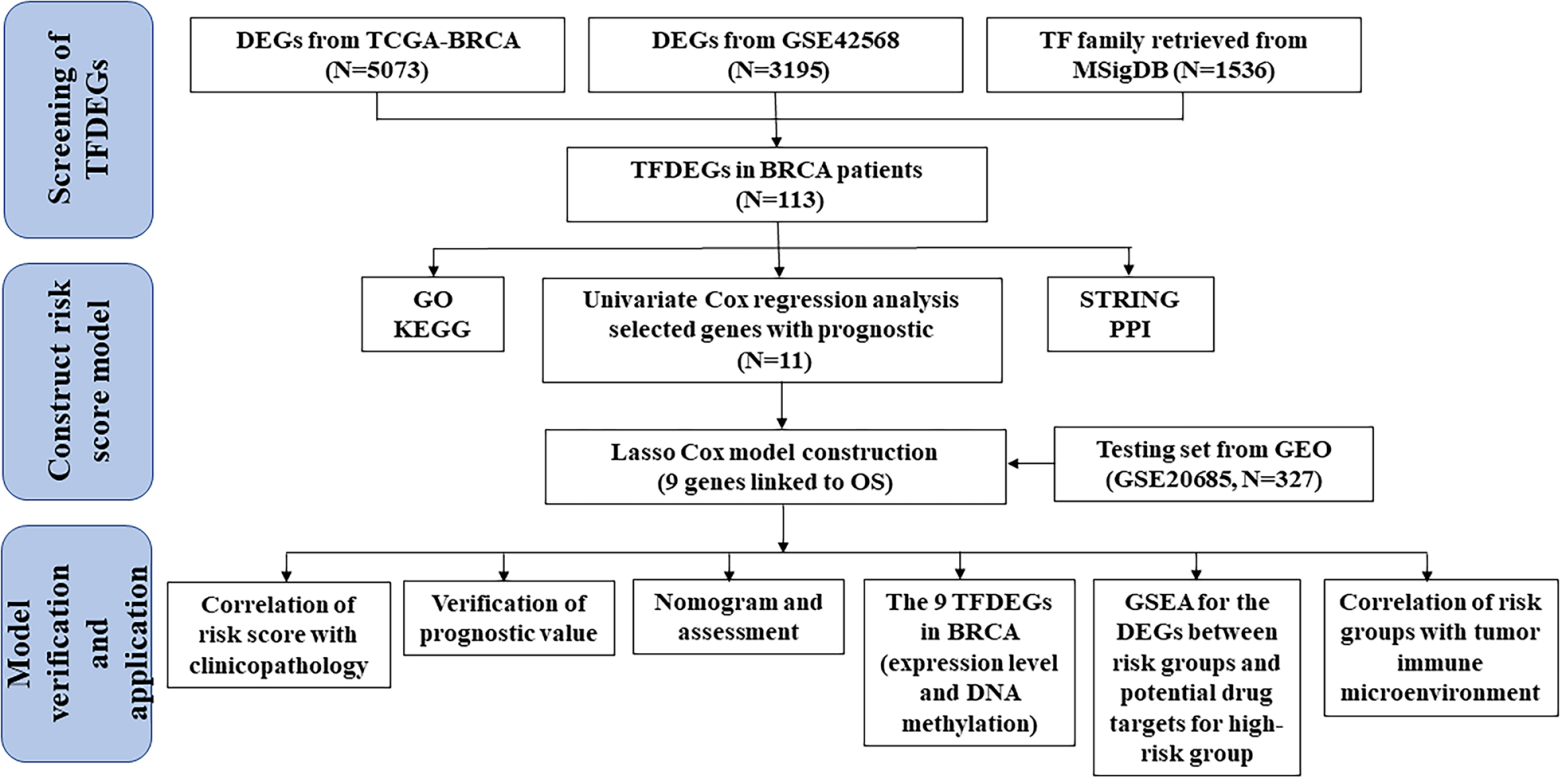
Flow diagram of the study

The detailed flowchart was shown in Fig. 1.

### Identification of TFDEGs in BRCA patients

**Fig. 2:**
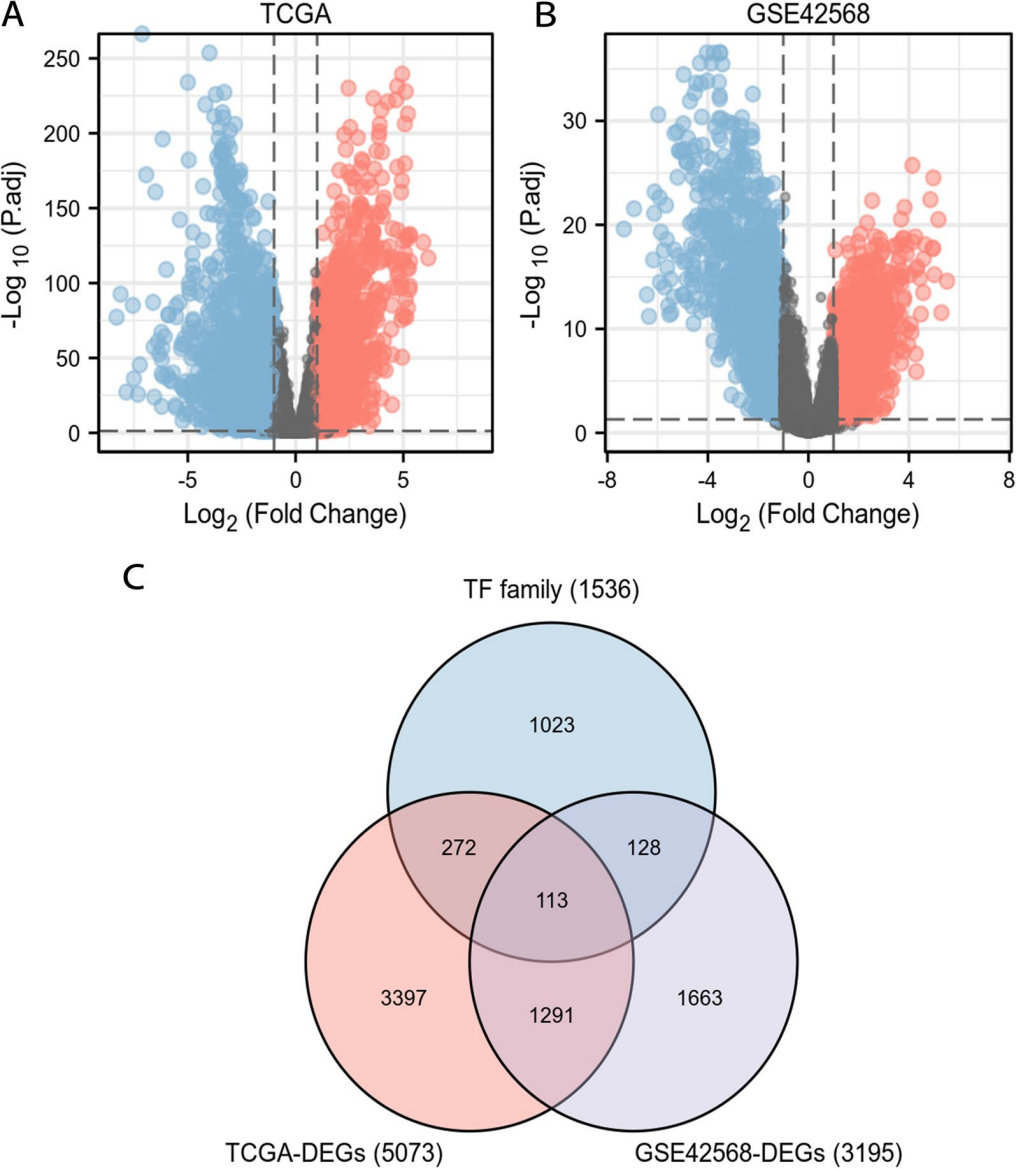
Screening of TFDEGs in BRCA. Volcano plots of differentially expressed genes (DEGs) analysis in set of **A** TCGA-BRCA and **B** GSE42568 (the red and blue dots are up- and down-regulated genes); **C** Venn analysis indicating the overlap of genes among DEGs in TCGA-BRCA, GSE42568 set and transcription factor (TF) family genes from Molecular Signatures Database (MSigDB)

The DEGs were collected from sets of TCGA-BRCA (Fig. 2A) and GSE42568 (Fig. 2B). According to the criteria, we obtained 5073 DEGs from TCGA-BRCA and 3195 DEGs from GSE42568, and downloaded 1536 genes of TF family on the basis of MSigDB (Additional file 2: Table S1). Based on overlapping DEGs among the aforementioned three algorithms, a total of 113 TFDEGs were screened for further analysis (Fig. 2C).

### Expression and functional clustering analysis of TFDEGs in BRCA

**Fig. 3:**
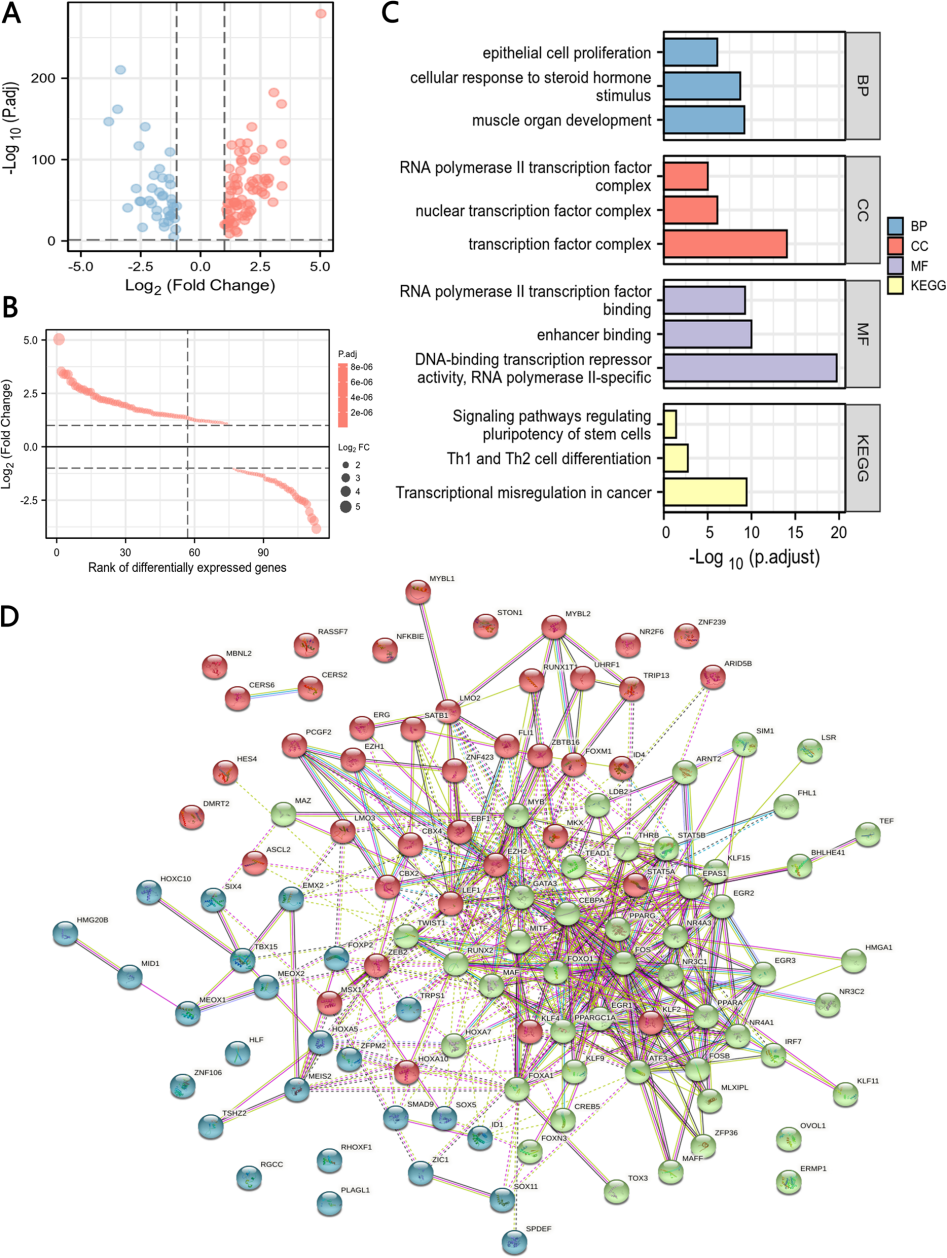
Expression and functional clustering analyses of 113 TFDEGs in BRCA. TFDEGs in TCGA-BRCA set displayed by: **A** Volcano Plot (the red and blue dots are up- and down-regulated genes) and **B** Differential Ranking Chart; **C** Gene ontology (GO) and Kyoto Encyclopedia of Genes and Genomes (KEGG) (www.kegg.jp/kegg/kegg1.html) analyses of TFDEGs; **D** protein–protein interaction (PPI) networks constructed by STRING

Volcano Plot (Fig. 3A) and Differential Ranking Chart (Fig. 3B) of the 113 TFDEGs (75 up-regulated and 38 down-regulated) were hereby performed based on TCGA-BRCA, with Go and KEGG pathway enrichment analyses to reveal the functions of the 113 TFDEGs. Moreover, these genes were obviously enriched in terms of epithelial cell proliferation, DNA-binding transcription, transcription factor complex, and transcriptional mis-regulation in cancer (Fig. 3C), while the PPI network among the 113 TFDEGs also be conducted (Fig. 3D).

### Constructing and validating risk score model

**Fig. 4:**
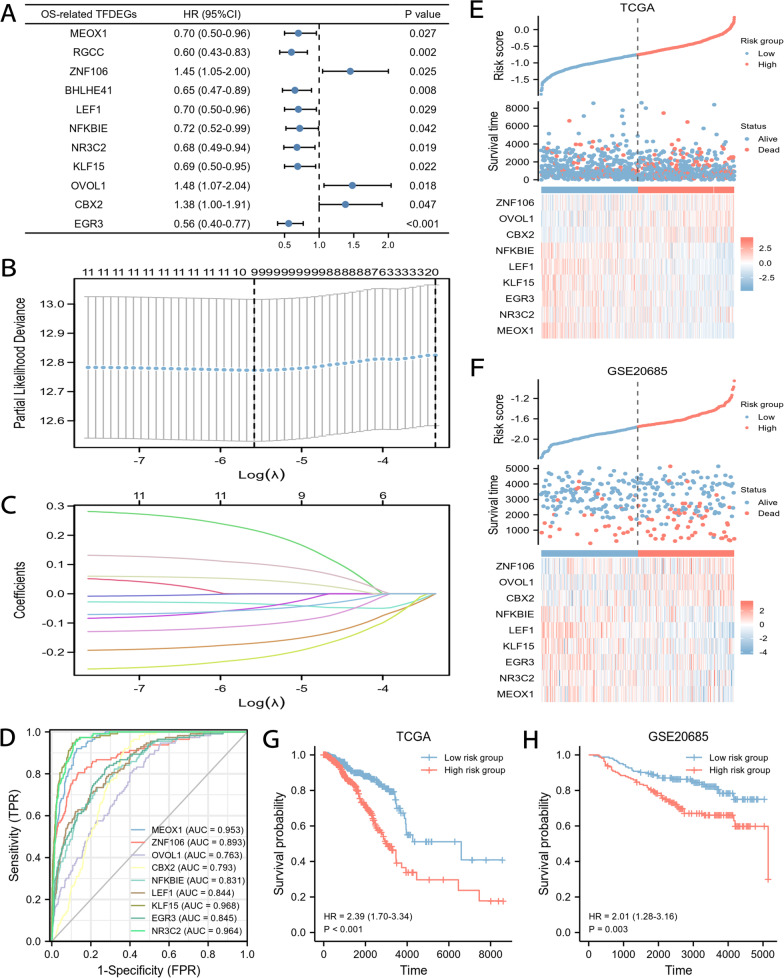
Construction and assessment of risk score model. **A** The 11 prognostic TFDEGs extracted by univariate Cox regression analysis and represented by a forest plot; **B** the tenfold cross-validation for variable selection in the LASSO model; **C** the LASSO coefficient profile of 11 TFDEGs; **D** receiver operating characteristic (ROC) curve suggesting the predictive precision of the risk score model; risk plot distribution, survival status of patients, and heat map including 9 TFDEGs in **E** TCGA-BRCA and **F** GSE20685; Kaplan–Meier (KM) survival curves of overall survival (OS) for patients between low-risk and high-risk groups in **G** TCGA-BRCA and **H** GSE20685

In this study, univariate Cox regression analysis was completed to explore the relationship between TFDEGs expression levels and OS in TCGA-BRCA. Eleven genes were identified as potential risk factors related to OS (Fig. 4A) by cut-off threshold of Cox *P* < 0.05, with the results of KM analyses shown in Additional file 3: Fig. S1. Furthermore, the LASSO regression algorithm was applied to refine the gene sets by calculating regression coefficients (Fig. 4B, C), after which 9 TFDEGs distinguished with most predictive value to establish the risk score model (Table 1). Based on the TFDEGs from the risk score model, the performance of ROC was then analyzed (Fig. 4D).

**Table 1:** TFDEGs and their relationship with OS, and their coefficients in LASSO regression model

Gene	Description	HR (95% CI)	*P* value	Coefficients
ZNF106 (ZNF474)	Zinc finger protein 106	1.45 (1.05–2.00)	0.025	0.217
OVOL1	Ovo-like zinc finger 1	1.48 (1.07–2.04)	0.018	0.103
CBX2	Chromobox homolog 2	1.38 (1.00–1.91)	0.047	0.048
NFKBIE	Nuclear factor of kappa light polypeptide gene enhancer in B-cells inhibitor, epsilon	0.72 (0.52–0.99)	0.042	− 0.221
LEF1	Lymphoid enhancer-binding factor 1	0.70 (0.50–0.96)	0.029	− 0.169
KLF15	Kruppel-like factor 15	0.69 (0.50–0.95)	0.022	− 0.108
EGR3	Early growth response 3	0.56 (0.40–0.77)	< 0.001	− 0.055
NR3C2	Nuclear receptor subfamily 3, group C, member 2	0.68 (0.49–0.94)	0.019	− 0.049
MEOX1	Mesenchyme homeobox 1	0.70 (0.50–0.96)	0.027	− 0.034

*TFDEGs* Differentially expressed transcription factor family genes; *OS* Overall survival; *LASSO* Least absolute shrinkage and selection operator; *HR* Hazard ratio; *95% CI* 95% confidence interval

The risk score for each patient in TCGA-BRCA (training set) and GSE20685 (testing set) was calculated in accordance with the expression levels of 9 genes and their regression coefficients, as shown in Fig. 4E and F respectively. After that, patients were divided into low-risk and high-risk groups by median risk score. According to general distribution of survival times, patients with higher risk score might bear worse OS (Fig. 4E, F). Furthermore, the expression levels of the screened genes were investigated in this study (Fig. 4E, F). As indicated in KM analyses, high-risk group witnessed a significantly poorer clinical outcome than those of low-risk group in both training (*P* < 0.001, Fig. 4G) and testing sets (*P* = 0.003, Fig. 4H), attesting to our risk score model’s great potential for predicting the prognosis of BRCA patients.

**Fig. 5:**
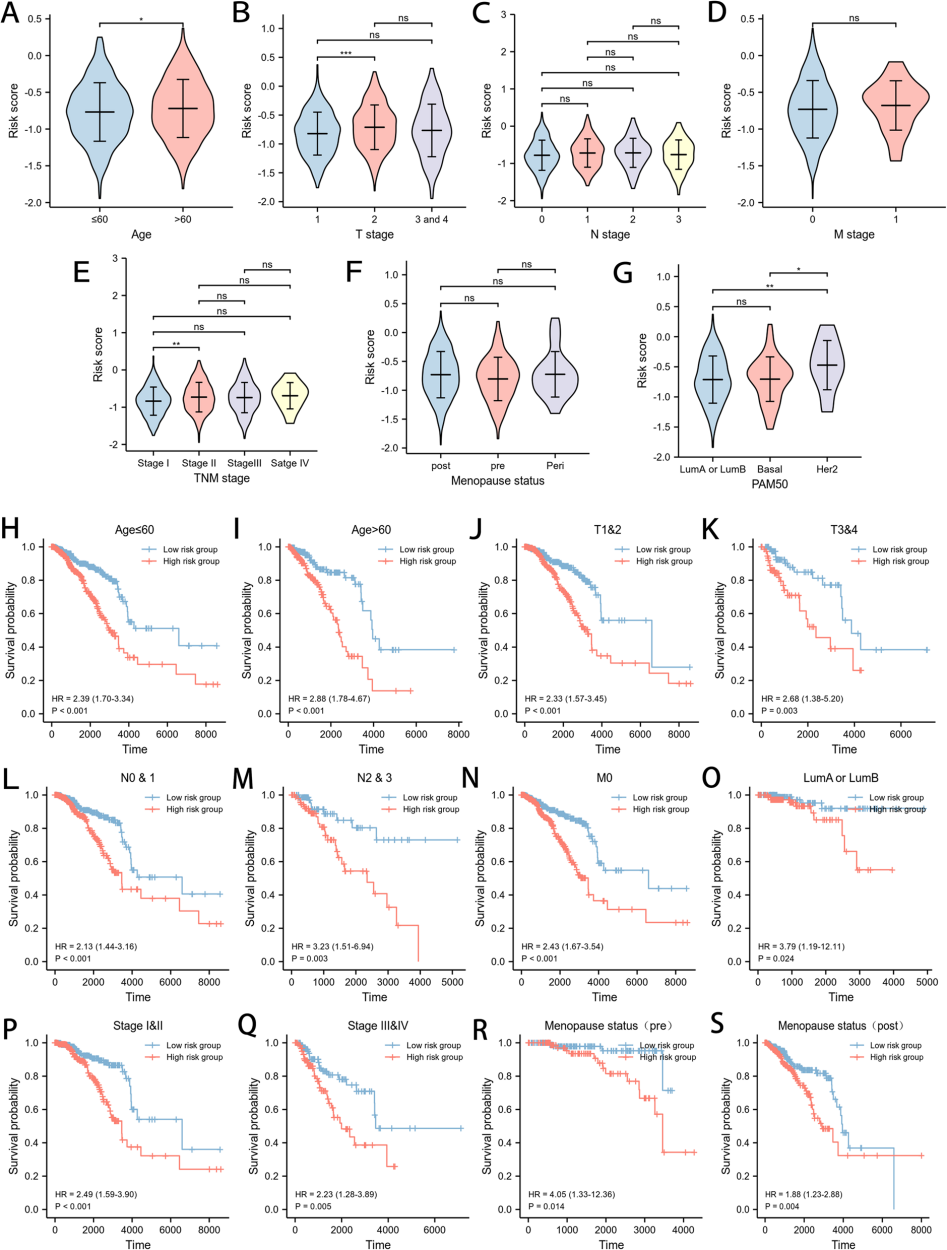
The predictive power of the risk score system. Correlation between the risk score and clinical characteristics: **A** age; **B** T stage; **C** N stage; **D** M stage; **E** TNM stage; **F** menopause status; **G** PAM50; KM curves for OS prediction of in subgroups of **H** age ≤ 60 years; **I** age > 60 years; **J** T1&2; **K** T3&4; **L** N0&1; **M** N2&3; **N** M0; **O** LumA or LumB; **P** stages I&II; **Q** stages III&IV; **R** menopause status (pre) and **S** menopause status (post)

As shown in Fig. 5A–G, the relationship between risk score and clinical characteristics in the training set (TCGA-BRCA) was analyzed in this study, suggesting obviously higher risk score in groups of patients with age > 60 years (*P* = 0.046), T stage: II (*P* < 0.001), TNM stage: II (*P* = 0.008), HER2 (compared with LumA or LumB: *P* = 0.005; compared with Basal: *P* = 0.020). Nevertheless, no obvious difference was found between the risk scores and N stage, M stage as well as menopause status.

In our study, the prediction efficiency of risk groups was further validated by several subgroups. According to the KM analyses, high-risk patients generally had a worse OS in subgroups of age ≤ 60 years, age > 60 years, T1&2, T3&4, N0&1, N2&3, M0, LumA or LumB, Stages I&II, Stages III&IV, menopause status (pre) and menopause status (post) (Fig. 5H–S). However, no significant difference was found in subgroups of M1 and Her2 & Basal (Additional file 4: Fig. S2).

### Nomogram

**Fig. 6:**
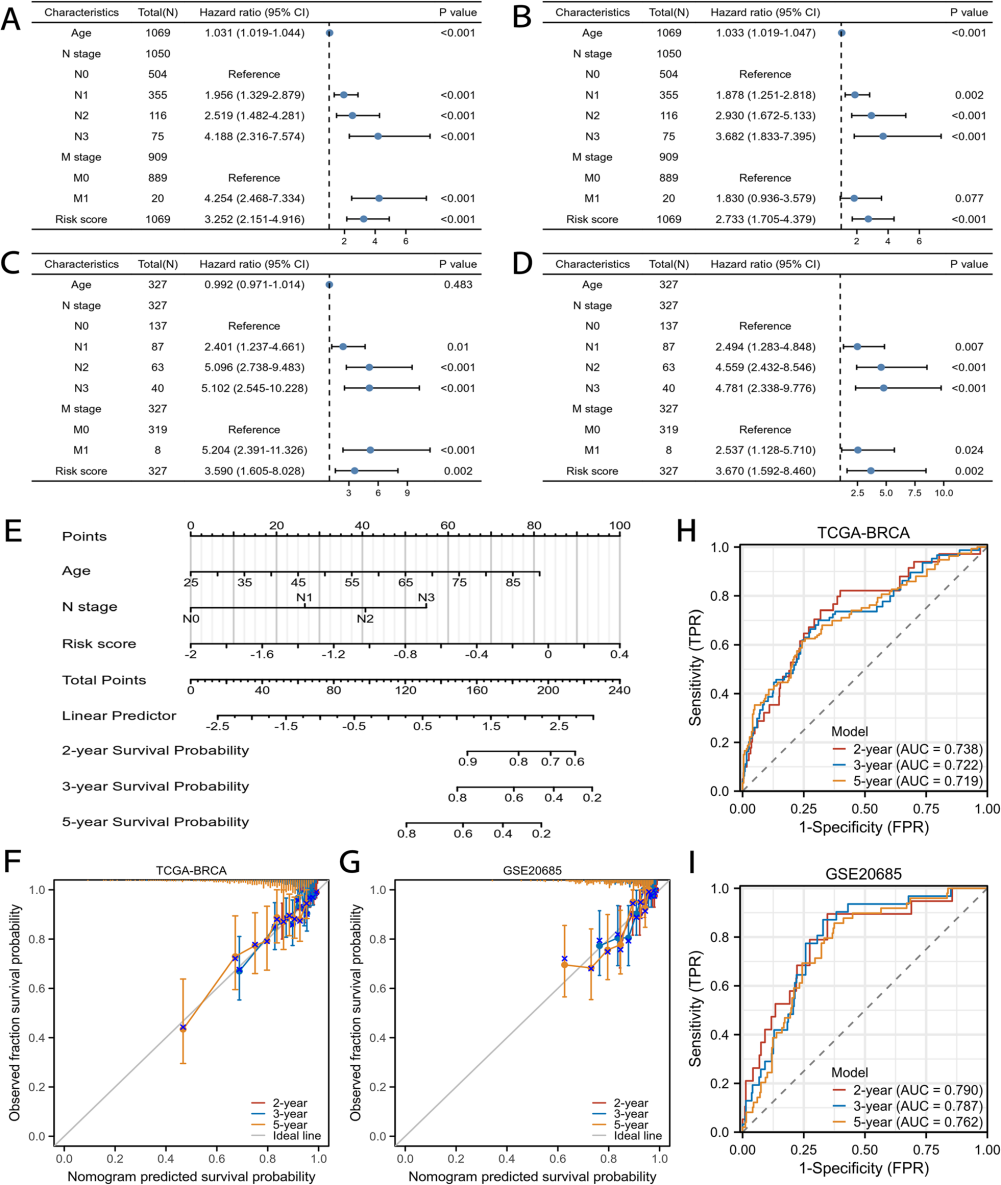
Nomogram and verification of prognostic model. **A** univariate and **B** multivariate Cox analyses of clinical factors and risk score with OS in TCGA-BRCA; **C** univariate and **D** multivariate Cox regression analyses of clinical factors and risk score with OS in GSE20685; **E** nomogram predicting 2-, 3- and 5-years survival rate of BRCA patients; the calibration curves for 2-, 3-, and 5-year OS in **F** TCGA-BRCA and **G** GSE20685; time-dependent ROC curves for predictive performance of the model in **H** TCGA-BRCA and **I** GSE20685

To determine whether the risk score model could be regarded as an independent risk factor for OS of BRCA patients, the potential prognostic indicators (age, T stage, N stage, M stage, ER status, PR status, HER2 status, and menopause status) were therefore analyzed via univariate and multivariate Cox regression in TCGA-BRCA (Table 2). For the purpose of in-depth study, all the independent prognostic clinicopathological parameters and risk scores were evaluated by univariate and multivariate Cox regression in TCGA-BRCA (Fig. 6A, B) and GSE20685 (Fig. 6C, D), indicating that high-risk group witnessed significantly worse OS in both training set [hazard ratio (HR) = 2.733, 95% confidence interval (95% CI) 1.705–4.379, *P* < 0.001] and testing set (HR = 3.670, 95% CI 1.592–8.460, *P* = 0.002). Therefore, the age, N stage and risk score were identified as independent risk factors for OS then integrated into nomogram model (Fig. 6E), with C-index (with corresponding 95% CI) of 0.712 (95% CI 0.685–0.739). After that, we calculated the score for each BRCA according, and the predictive capability and consistency of the nomogram were assessed by calibration curve. The calibration plots showed outstanding consistency among the 2-, 3-, and 5-year OS rates when comparing with the ideal and nomogram model in both training (Fig. 6F) and testing (Fig. 6G) sets. Besides, time-dependent ROC analysis was also utilized to assess the discriminatory ability of the nomogram (Fig. 6H, I).

**Table 2:** Univariate and multivariate Cox analysis of OS in TCGA-BRCA

Characteristics	Total (N)	Univariate analysis		Multivariate analysis	
		HR (95% CI)	*P* value	HR (95% CI)	*P* value
Age	1082				
≤ 60	601	Reference			
> 60	481	2.020 (1.465–2.784)	** < 0.001**	3.154 (1.616–6.155)	** < 0.001**
T stage	1079				
T1	276	Reference			
T2	629	1.332 (0.887–1.999)	0.166	1.013 (0.520–1.973)	0.970
T3&T4	174	1.953 (1.221–3.123)	**0.005**	1.649 (0.683–3.982)	0.266
N stage	1063				
N0	514	Reference			
N1	357	1.956 (1.329–2.879)	** < 0.001**	1.376 (0.702–2.697)	0.352
N2	116	2.519 (1.482–4.281)	** < 0.001**	2.985 (1.297–6.869)	**0.010**
N3	76	4.188 (2.316–7.574)	** < 0.001**	6.068 (2.364–15.573)	** < 0.001**
M stage	922				
M0	902	Reference			
M1	20	4.254 (2.468–7.334)	** < 0.001**	3.639 (1.152–11.498)	**0.028**
ER status	1032				
Negative	240	Reference			
Positive	792	0.712 (0.495–1.023)	0.066	0.469 (0.189–1.164)	0.102
PR status	1029				
Negative	342	Reference			
Positive	687	0.732 (0.523–1.024)	0.068	1.028 (0.440–2.403)	0.948
HER2 status	715				
Negative	558	Reference			
Positive	157	1.593 (0.973–2.609)	0.064	0.931 (0.476–1.821)	0.836
Menopause status	931				
Pre	229	Reference			
Post	702	2.165 (1.302–3.600)	**0.003**	2.083 (0.863–5.032)	0.103

*BRCA* Breast cancer; *TCGA* The Cancer Genome Atlas; *OS* Over survival; *HR* Hazard Ratio; *95% CI* 95% confidence interval; *ER* Estrogen receptor; *PR* Progesterone receptor; *HER2* Human epidermal growth factor receptor 2. Bold indicates that the difference is statistically significant

### The DEGs between groups and potential drug targets for high-risk group

**Fig. 7:**
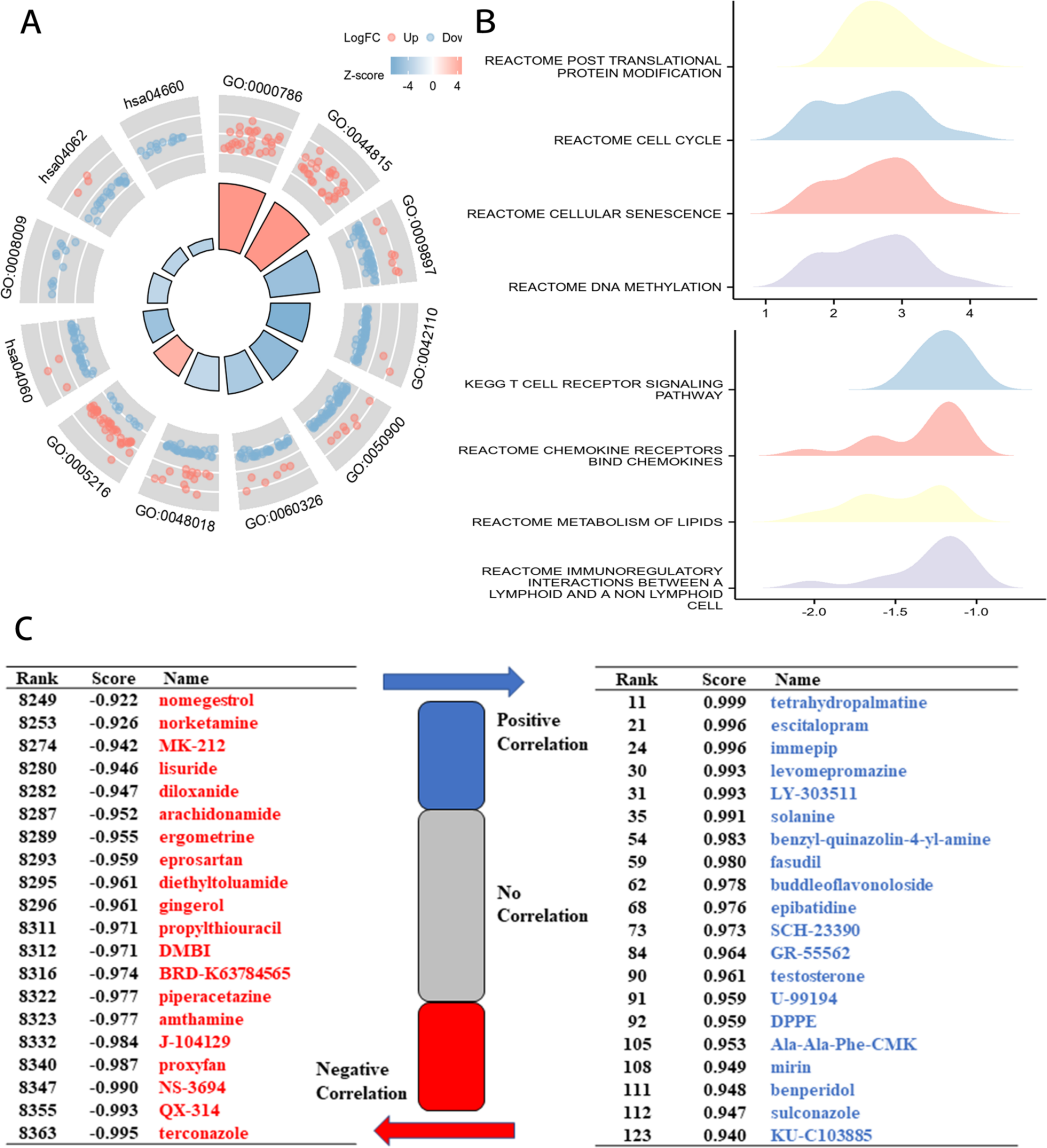
The DEGs between groups and potential drug targets for high-risk group. **A** GO and KEGG analyses of DEGs from high-risk group compared with low-risk group; **B** ridge map of gene set enrichment analysis (GSEA) for DEGs from high-risk group compared with low-risk group; **C** Connectivity Map (CMap) analysis to find the potential drug targets for high-risk group. The 20 top and bottom drugs represented positive and negative correlations, respectively, with BRCA patients in high-risk group. CMap analysis identified terconazole, QX-314, and several histamine receptor modulators as potential therapeutic drugs for BRCA patients in high-risk group

To clarify the potential influence of the screened TFDEGs expression levels on transcriptomic profile of BRCA, Go and KEGG pathway enrichment analyses were performed in order to investigate the functions of DEGs from high-risk group, comparing with low-risk group in TCGA-BRCA (Fig. 7A), suggesting these genes significantly enriched in terms of T cell activation, cell chemotaxis, receptor ligand activity together with ion channel activity. Based on GSEA analysis of DEGs from high-risk group compared with low-risk group, pathways as those related to cell cycle, cellular senescence and DNA methylation were enriched in high-risk group, while T cell receptor signaling pathway, metabolism of lipids, chemokine receptors bind chemokines, and immunoregulatory interactions were enriched in low-risk group (Fig. 7B), indicating TFDEGs' potential role in the metabolism, progression and tumor microenvironment of BRCA.

Besides, we uploaded both up- and down-regulated DEGs into the CMap database to predict potential drug the for high-risk group. CMap applies a systematic approach to reveal interactions among drugs, compounds, and diseases based on DEGs from high-risk group compared with low-risk group. The top 20 drugs with positive correlations and the top 20 drugs with negative correlations were obtained from CMap (Fig. 7C). These drugs were ranked by p-values and were determined based on the DEGs signatures against the CMap database. Terconazole, QX-314, and several histamine receptor modulators may serve as potential therapeutic drugs for BRCA patients in high-risk group.

### Analyses of 9 TFDEGs in risk score model

**Fig. 8:**
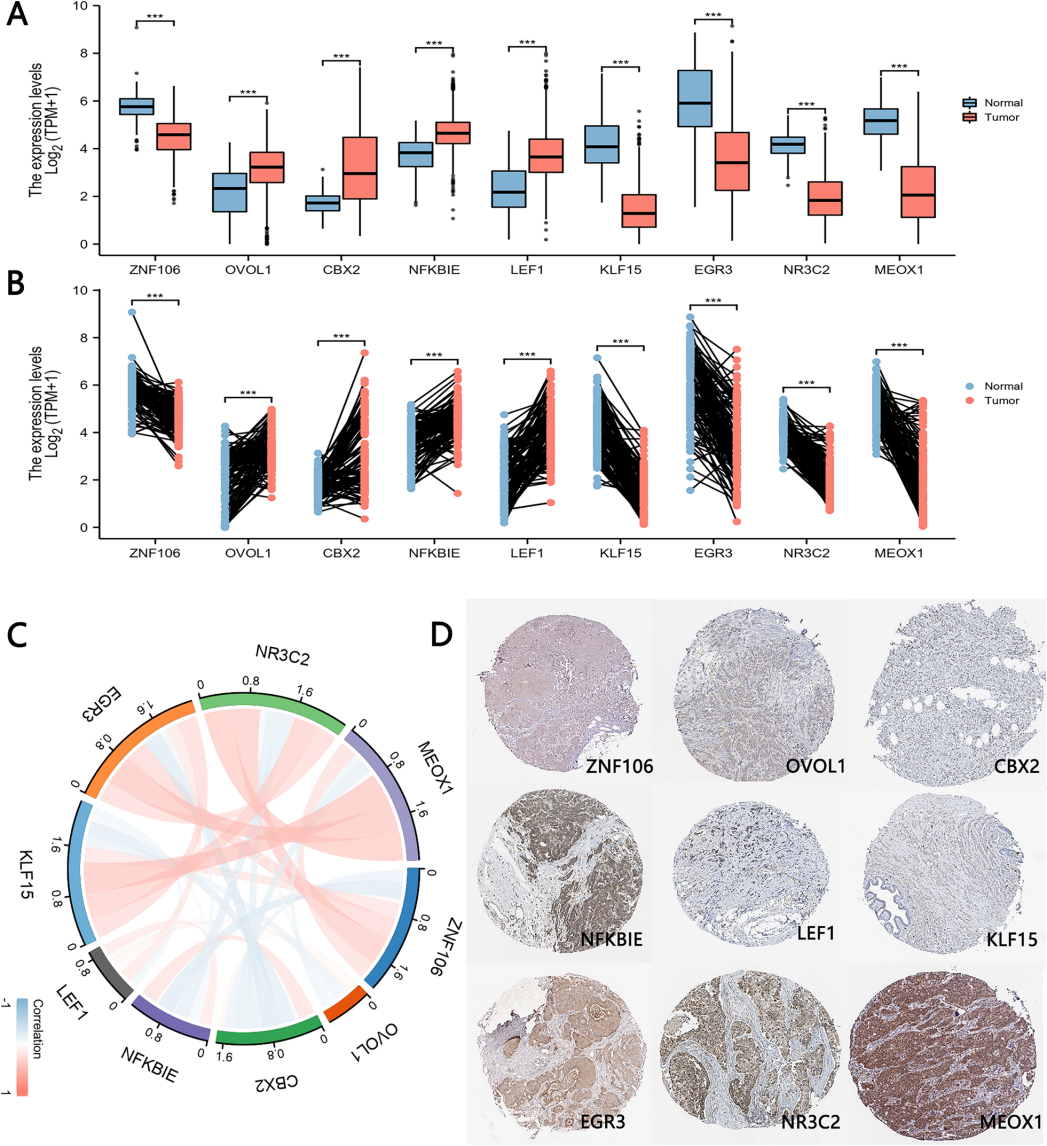
Analyses of 9 TFDEGs in prognostic model. Expression of 9 TFDEGs between normal and tumor in TCGA-BRCA by **A** unpaired samples and **B** paired samples; **C** relationship among the 9 TFDEGs; **D** the protein expression of 9 TFDEGs in BRCA tissue by immunohistochemistry from HPA database

The 9 TFDEGs were significantly differential expression (4 up-regulated and 5 down-regulated) in TCGA-BRCA (Fig. 8A, B) and closely related to each other (Fig. 8C). In addition, immunohistochemical analysis in HPA was applied to further investigate the expression level of the 9TFDEGs (Fig. 8D).

DNA methyltransferases on CpG island methylation are transcription factors in the suppression or promotion of cell growth which is a reversible process [31]. In this study, we present the heatmap and prognostic value of DNA methylation clustering the expression levels of the 9 TFDEGs in BRCA (Additional file 5: Fig. S3 and Additional file 6: Table S2). With regard to DNA methylation expression levels, cg01024618, cg05008688, cg07787851 from ZNF106; cg16233472, cg19694099, cg15453482 from OVOL1; cg22228071, cg18045515, cg22892904, cg07335357, cg14726117, cg17346145 from CBX2; cg19109431 from NFKBIE; cg12271317, cg00337658, cg11113607 from LEF1; cg07275757, cg10590842 from NR3C2 came up with the highest levels and significant prognostic values (likelihood ratio (LR) test *P* < 0.05) in BRCA.

### Correlation between prognostic model and TIME

**Fig. 9:**
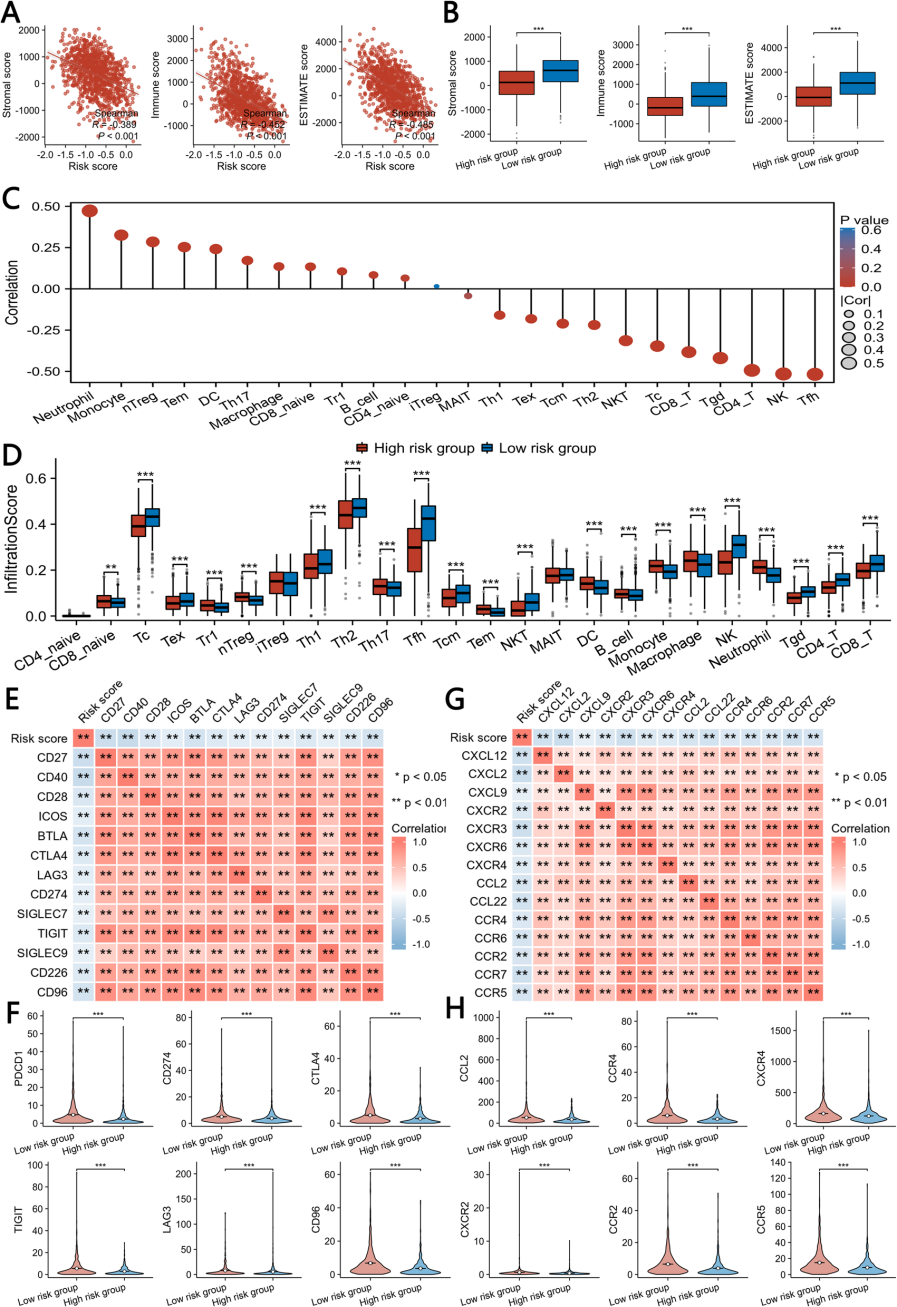
Correlation between prognostic model and TIME. **A** Relationship between immune infiltration scores (including Stromal score, Immune score, and ESTIMATE score) and risk score; **B** comparison of immune infiltration scores (including Stromal score, Immune score, and ESTIMATE score) between low-risk and high-risk groups; **C** correlations between risk model and tumor-infiltrating immune cells (TIICs); **D** comparisons of TIICs between low-risk and high-risk groups; **E** association between risk score and immune checkpoints; **F** comparison of six immune checkpoints between low-risk and high-risk groups; **G** association between risk score and chemotactic factors; **H** comparison of six chemotactic factors between low-risk and high-risk groups

Based on this study, the risk score was negatively correlated with immune infiltration score (including Stromal score, Immune score, and ESTIMATE score) (Fig. 9A, B).

In addition, the correlation between the prognostic model and immune cells infiltration of patients in TCGA-BRCA cohort were also taken into account, with the proportion of different infiltrating immune cells retrieved from ImmuCellAI. The results indicated that the risk score was positively correlated with neutrophil, monocyte, nTreg, Tem, DC, and Th17, while negatively correlated with Tfh, NK, CD4 + T cells, Tgd, CD8 + T cells, Tc, and NKT (Fig. 9C). Furthermore, the high-risk group generally witnessed less infiltrating immune cells, especially CD4 + and CD8 + T cells (Fig. 9D).

As analyzed with the TIMER tool, expressions of TFDEGs were also correlated with immune infiltration profiles in BRCA. In summary, expression of each TFDEGs gene was associated with tumor purity and markers of different immune cells (Additional file 7: Fig. S4).

Moreover, we evaluated the relationship between risk score model and immune checkpoints (Fig. 9E), to find the expression levels of PDCD1 (PD-1), CD274 (PD-L1), CTLA4, LAG3, TIGIT and CD96 negatively correlated with the risk score (Fig. 9F).

Finally, we analyzed the relationship between risk score model and chemotactic factors (Fig. 9G), suggesting that the expression levels of CCL2, CCR4, CXCR4, CCR2, CCR5 and CXCR2 were negatively correlated with the risk score (Fig. 9H).

## Discussion

To our knowledge, BRCA lists as the leading cause of cancer-related mortality among women all over the world. Despite of remarkable progress in diagnosis and treatment of BRCA over the last decades, a wide array of problems regarding its progression, metastasis and treatment resistance are yet to be fully clarified [32–34].

TFs recognize specific DNA sequences to control chromatin and transcription, forming a complex system to guide the genome expression [9]. With a key role in human physiology, disease and variation, TFs are closely related to the occurrence and development of tumors [35–37].

In recent years, an increasing number of studies have found several TFs to participate in tumorigenesis, progression together with the microenvironment of BRCA, indicating their potential and promising role to serve as prognostic markers for BRCA [38–41]. Nevertheless, they appeared to be inconsistent due to small datasets, the heterogeneity of BRCA, as well as variation in data pre-processing approaches. It is worth noting that several studies have figured out multigene panels' possible role as prognostic indicators in BRCA [42–46], while all these studies only focused on the identification of prognostic signatures. Hence, this study targeted at a comprehensive profiling of TF family in BRCA patients, in order to develop a prognostic model and explore its correlation with TIME.

In this study, we obtained gene expression and clinicopathological data of BRCA from TCGA and GEO database, after which, by means of univariate Cox analysis and LASSO regression analysis, a risk score model composed of nine genetic biomarkers was therefore established based on TFDEGs. Furthermore, the risk score was verified to be both effective and stable for predicting the prognosis of low-risk and high-risk groups in TCGA-BRCA and subgroups via KM curve. In order to predict the OS of BRCA patients, we also established a prognostic nomogram model based on TFDEGs, with a comprehensive integration of the risk score, age and N stage. Calibration plots revealed the robust predictive ability of the prognostic nomogram for OS in TCGA and GSE20685 cohorts, suggesting our prognostic model's great potential in predicting the clinical outcomes of BRCA patients.

The 9-TFDEGs prognostic model was comprised of ZNF106, OVOL1, CBX2, NFKBIE, LEF1, KLF15, EGR3, NR3C2 and MEOX1, all of which were protein coding gene. In BRCA, ZNF106, KLF15, EGR3, NR3C2 and MEOX1 were significantly down-regulated, while OVOL1, CBX2, NFKBIE, LEF1 were obviously up-regulated.

With a variety of cellular functions, including insulin receptor signaling, rRNA transcriptional regulation, ZNF106 is essential for maintaining motor and sensory neurons [47]. According to Guo et al. [48] ZNF106 enjoyed great prognostic significance in tumors with considerably lower expression. OVOL1, a key mediator of epithelial lineage determination and mesenchymal–epithelial transition (MET), has been proved to be inversely correlated with the epithelial-mesenchymal transition (EMT) signature and serve as a great prognostic indicator for BRCA patients [49]. In addition, CBX2 encodes a component of the multiprotein complex, which is required to maintain the transcriptionally repressive state of many genes throughout development via chromatin remodeling and modification of histones. Furthermore, Iqbal et al. [50] have reported that CBX2 and CBX7 could predict the outcomes and sensitivity to FDA-approved/investigational drugs in BRCA, and pathways related to NFKBIE could lead to the activation of NF-KappaB by PKR and bacterial infections in CF airways. Study had revealed that it might be associated with the potential target of triple-negative breast cancer (TNBC) [51]. Moreover, the protein encoded by LEF1 can bind to a functionally important site in the T-cell receptor-alpha enhancer, thereby conferring maximal enhancer activity. As a TF of EMT, it is involved in the Wnt signaling pathway. Apart from EMT, LEF1 facilitates metastasis by improving the antioxidative capacity of epithelial BRCA cells [52]. As a putative BCCA suppressor gene, KLF15 was recorded to get involved in negative regulation of peptidyl-lysine acetylation, together with positive regulation of transcription by RNA polymerase II [53]. The protein of EGR3 encoded by this gene participates in the transcriptional regulation of genes in controlling biological rhythm, and it may also play a certain role in various processes including muscle development, lymphocyte development, endothelial cell growth and migration, as well as neuronal development. Furthermore, Inoue has found EGR3 involved in the estrogen-signaling pathway and closely associated with the genesis and malignant progression of BRCA [54]. The NR3C2 protein functions as a ligand-dependent TF binding to mineralocorticoid response elements in order to transactivate target genes. As reported, miR-301b may be a tumor-promoting miRNA in BRCA, and miR-301b/NR3C2 axis mediated tumor development from cell proliferation and migration [55]. Additionally, study had revealed that combined p53- and PTEN-deficiency in TNBC activated expression of the MEOX1, which might serve as a potential therapeutic target for managing p53- and PTEN-deficient TNBC [56].

Moreover, we performed GO and KEGG functional analyses based on DEGs between low-risk and high-risk groups, indicating that DEGs were significantly enriched in terms of T cell activation, cell chemotaxis, receptor ligand activity and ion channel activity, which were closely related to the formation and development of tumors and immune microenvironment. Therefore, we further conducted GSEA between low-risk and high-risk groups, suggesting that pathways associated with formation and progression of tumor were enriched in the high-risk group, while pathways related to tumor associated immune cells and immunotherapeutic responses regulation were enriched in the low-risk group.

In addition, through CMap analysis, we revealed that certain drugs with highly negative correlations might serve as a potential treatment for high-risk group. Of course, this result needs to be further verified in vitro and vivo. Terconazole (TCZ), an azole antifungal drug, has been shown to enhance the cytotoxicity of antimitotic drugs in P-glycoprotein-overexpressing-resistant cancer cells [57]. Fuseya et al. [58] have found that systemic administration of QX-314 in mice could inhibit some behavioral aspects of bone cancer pain through selective inhibition of TRPV1-expressing afferents without coadministration of TRPV1 agonists. Cell proliferation is critical for tumor development and progression, and histamine is a main mediator of this biological process in different types of cancers. It has been recorded that histamine receptor modulator could regulate cancer-associated biological processes during cancer development in multiple cell types, including neoplastic cells and cells in the tumor micro-environment [59]. Collectively, the results suggested that these drugs are potential therapeutic drugs for high-risk group.

Several studies have figured out that TIME was correlated with the prognosis of cancer patients [60–63]. To further clarify the relationship between TIME and the prognostic model, analysis was hereby performed to reveal the correlation between risk score and ESTIMATE score, tumor infiltrating immune cells, immune checkpoints as well as chemotactic factors.

In immune infiltration analyses, the risk score was found to be negatively correlated with ESTIMATE score, composing of Stromal score and Immune score. With regard to tumor infiltrating immune cells analysis, the risk score was negatively correlated with infiltration levels of CD4+ and CD8+T cells, which played a key role in immunotherapy [64, 65]. Based on immune checkpoints and chemotactic factors analyses, the established risk score was negatively correlated with the expression levels of immune checkpoints (such as PD1, PD–L1, CTLA4, LAG3, TIGIT, etc.) and chemotactic factors (including CCL2, CCR4, CXCR4, CCR2, CCR5, etc.). In summary, compared with BRCA in low-risk groups, tumors of high-risk groups tended to be immunologically “cold” and might not benefit from immunotherapy.

**Fig. 10:**
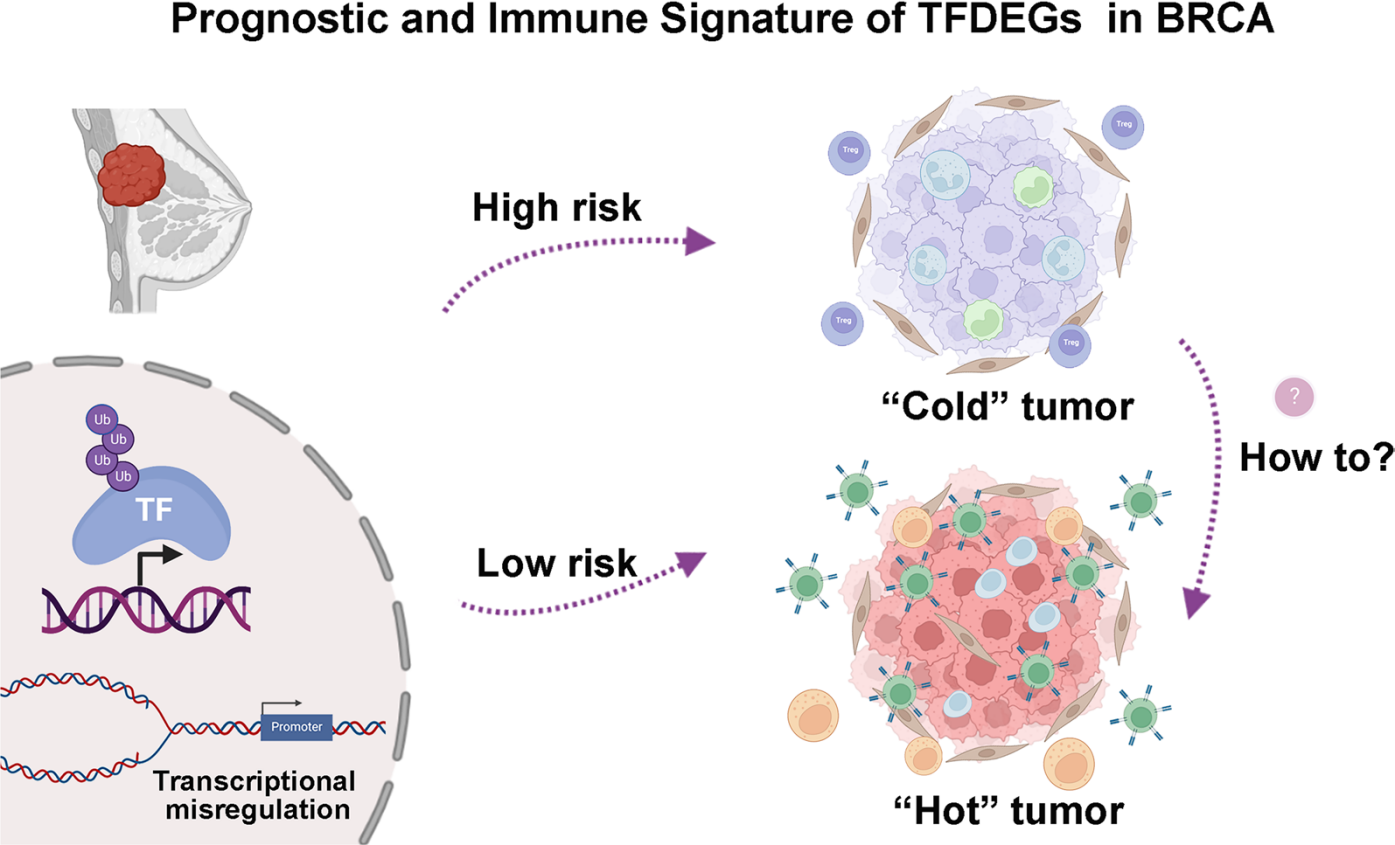
A model for prognostic and immune signature of TFDEGs in BRCA

In conclusion, the prognostic model based on TFDEGs can well predict the prognosis of BRCA patients; moreover, it may also be utilized to screen the appropriate immunotherapy benefit population and predict potential drug targets. The top of agenda for future research is to precisely identify high-risk groups, reverse the occurrence and development of tumor by screening effective drug targets, and transform immunologically “cold” tumors into responsive “hot” lesions by changing the TIME (Fig. 10) [66–68].

## Conclusions

The prognostic model based on TFDEGs could distinguish as a novel biomarker for predicting prognosis of BRCA patients; in addition, it may also be utilized to identify potential benefit population from immunotherapy in different TIME and predict potential drug targets.

## Supplementary Information

**Additional file 1.** R code and data.

**Additional file 2. Table S1.** The TF family genes.

**Additional file 3: Figure S1.** Kaplan–Meier curves for OS prediction in TCGA-BRCA of A MEOX1; B RGCC; C ZNF106; D BHLHE41; E LEF1; F NFKBIE; G NR3C2; H KLF15; I OVOL1; J CBX2; K EGR3.

**Additional file 4: Figure S2.** Kaplan–Meier curves for OS prediction in BRCA subtypes of A Her2 or Basal and B M1.

**Additional file 5: Figure S3.** Heatmap of DNA methylation expression levels of the TFDEGs in BRCA by MethSurv platform.

**Additional file 6: Table S2.** Prognostic Value of Single CpG of the TFDEGs in BRCA by MethSurv platform.

**Additional file 7: Figure S4.** Correlations between expressions of TFDEGs and immune infiltration profiles in BRCA. The figure shows the expression of each gene associated with tumor purity and several tumor-infiltrating immune cell markers, such as B cell, CD8+ T cell, CD4 + T cell, macrophage, neutrophil, and dendritic cell markers.

## Data Availability

The data sets used and/or analyzed in this study are available from the cancer genome database (TCGA-BRCA) (https://portal.gdc.cancer.gov), NCBI Gene Expression Omnibus (GEO: datasets of GSE42568 and GSE20685) (https://www.ncbi.nlm.nih.gov/geo/), Molecular Signatures Database (MSigDB) (http://www.gsea-msigdb.org/gsea/msigdb/index.jsp), KEGG pathway database (www.kegg.jp/kegg/kegg1.html), Connectivity Map (CMap) database (https://clue.io), Human Protein Atlas (HPA) database (http://www.proteinatlas.org/): HPA054267 (patient id: 4193), HPA003984 (patient id: 2091), HPA023083 (patient id: 2805), HPA005941 (patient id: 1939), CAB019405 (patient id: 2898), HPA028866 (patient id: 2091), HPA006206 (patient id: 2428), CAB009155 (patient id: 2015), HPA045214 (patient id: 2160), MethSurv database (https://biit.cs.ut.ee/methsurv/), and ImmuCellAI (http://bioinfo.life.hust.edu.cn/ImmuCellAI) database. Besides, R code, data input, and output of this study were provided in Additional file 1: R code and data.

## Abbreviations

TFDEGs: Differentially expressed transcription factor family genes
DEGs: Differentially expressed genes
TF: Transcription factor
MSigDB: Molecular Signatures Database
GO: Gene ontology
KEGG: Kyoto Encyclopedia of Genes and Genomes
PPI: Protein–protein interaction
KM: Kaplan–Meier
GSEA: Gene set enrichment analysis
BRCA: Breast cancer
TCGA: The Cancer Genome Atlas
GEO: Gene Expression Omnibus
LASSO: Least Absolute Shrinkage and Selection Operator
OS: Overall survival
STRING: Search Tool for the Retrieval of Interacting Genes
ROC: Receiver operating characteristic
HPA: Human Protein Atlas
C-index: Concordance index
ER: Estrogen receptor
PR: Progesterone receptor
HER2: Human epidermal growth factor receptor 2
HR: Hazard ratio
CIs: Confidence intervals
TNBC: Triple-negative breast cancer
TIME: Tumor immune microenvironment
TIICs: Tumor-infiltrating immune cells
CMap: Connectivity map
ImmuCellAI: Immune Cell Abundance Identifier
TIMER: Tumor Immune Estimation Resource
LR: Likelihood ratio
