# Supplementary material for: Comprehensive research into prognostic and immune signatures of transcription factor family in breast cancer
Source: BMC Med Genomics. 2023 Apr 25;16:87. doi: 10.1186/s12920-023-01521-y (PMC10127334; doi:10.1186/s12920-023-01521-y)
Supplement: Supplementary file 1 — Additional file 1. R code and data. [file 12920_2023_1521_MOESM1_ESM.zip › Supplementary R code and data/CMap/process.docx]

https://clue.io

chose “Tools” “Query” “Gene expression(L1000)”

input genes

submit

chose “DETAILED LIST” “Compound×2425” “Export”
