# Supplementary material for: Comprehensive research into prognostic and immune signatures of transcription factor family in breast cancer
Source: BMC Med Genomics. 2023 Apr 25;16:87. doi: 10.1186/s12920-023-01521-y (PMC10127334; doi:10.1186/s12920-023-01521-y)
Supplement: Supplementary file 1 — Additional file 1. R code and data. [file 12920_2023_1521_MOESM1_ESM.zip › Supplementary R code and data/PPI/process.docx]

<https://cn.string-db.org>

chose “multiple proteins”

113 input genes

chose “Homo sapiens”

search
