# Supplementary figures and images for: Comprehensive research into prognostic and immune signatures of transcription factor family in breast cancer
Source: BMC Med Genomics. 2023 Apr 25;16:87. doi: 10.1186/s12920-023-01521-y (PMC10127334; doi:10.1186/s12920-023-01521-y)

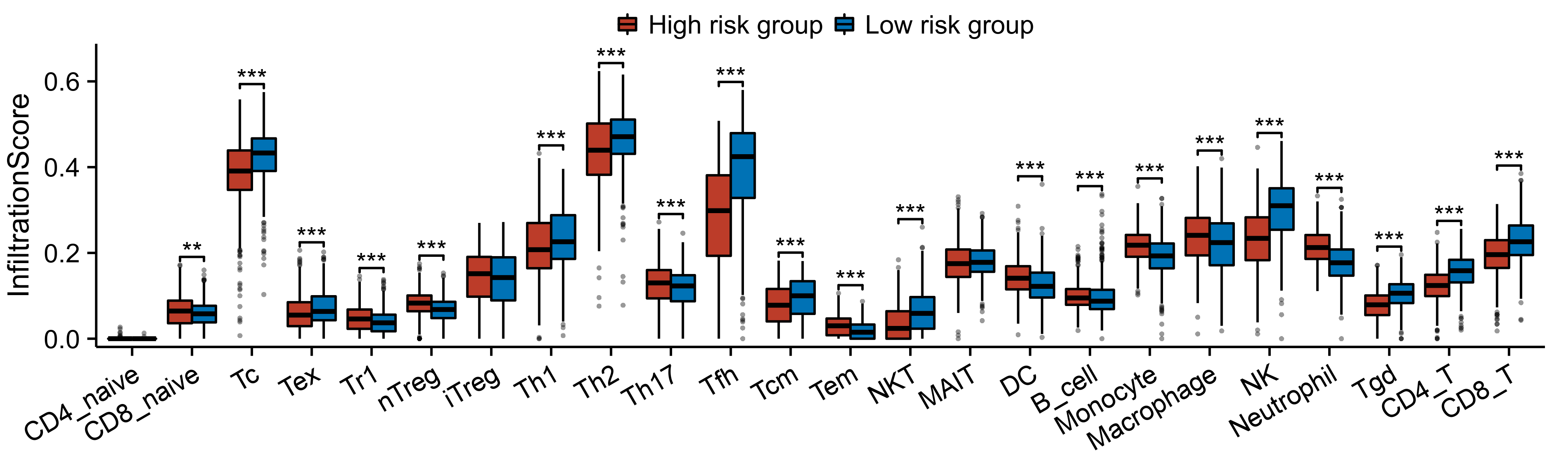

Supplement: Supplementary file 1 — Additional file 1. R code and data. [file 12920_2023_1521_MOESM1_ESM.zip › Supplementary R code and data/ImmucellAI/immune.diff.tiff]

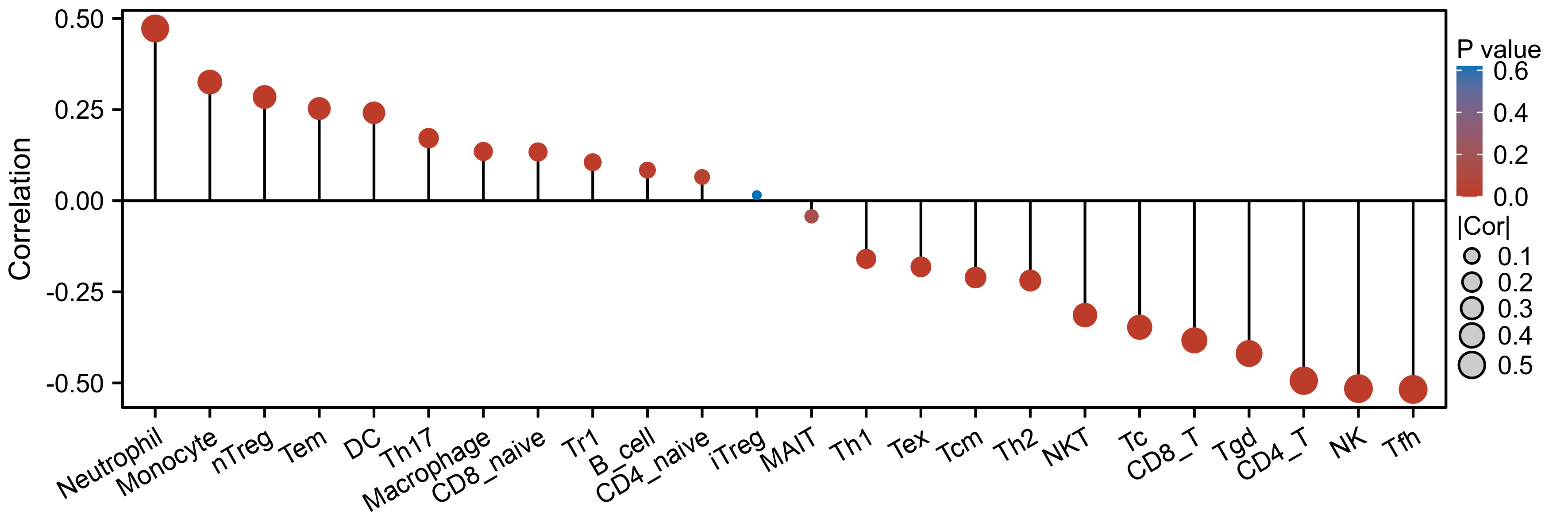

Supplement: Supplementary file 1 — Additional file 1. R code and data. [file 12920_2023_1521_MOESM1_ESM.zip › Supplementary R code and data/ImmucellAI/Lollipop.tiff]

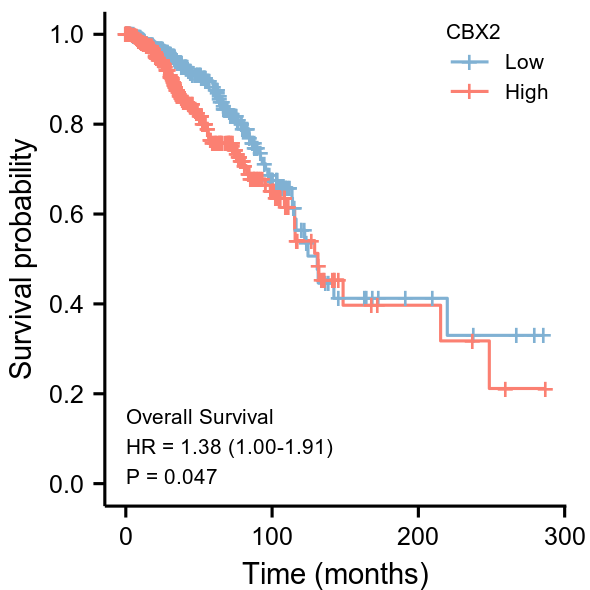

Supplement: Supplementary file 1 — Additional file 1. R code and data. [file 12920_2023_1521_MOESM1_ESM.zip › Supplementary R code and data/KM curve/CBX2.survival.tiff]

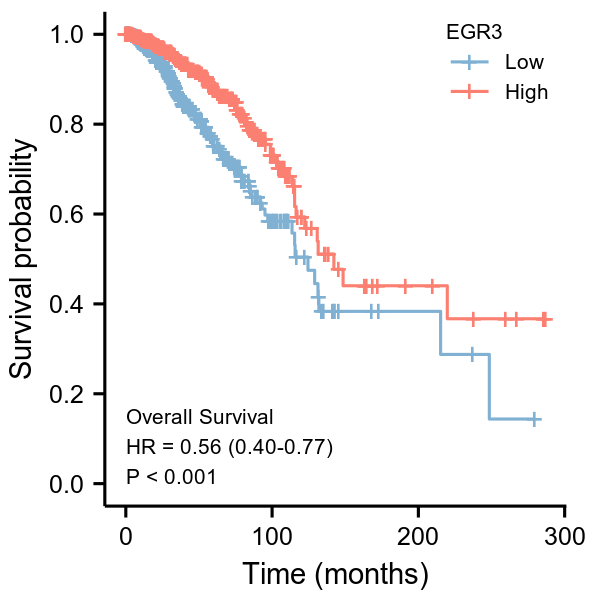

Supplement: Supplementary file 1 — Additional file 1. R code and data. [file 12920_2023_1521_MOESM1_ESM.zip › Supplementary R code and data/KM curve/EGR3.survival.tiff]

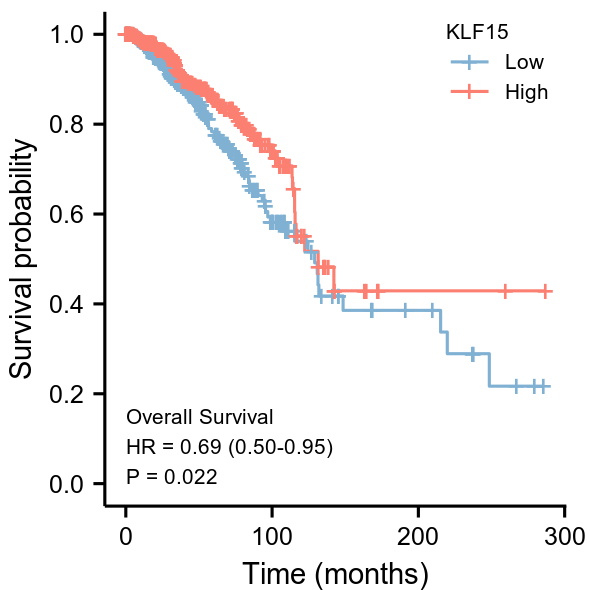

Supplement: Supplementary file 1 — Additional file 1. R code and data. [file 12920_2023_1521_MOESM1_ESM.zip › Supplementary R code and data/KM curve/KLF15.survival.tiff]

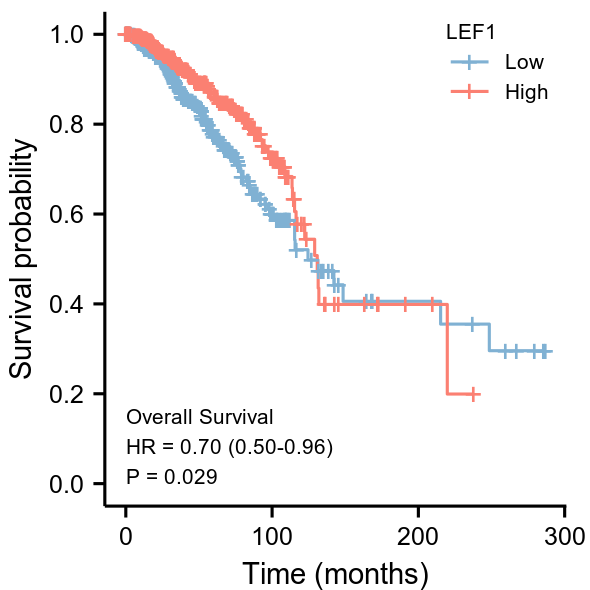

Supplement: Supplementary file 1 — Additional file 1. R code and data. [file 12920_2023_1521_MOESM1_ESM.zip › Supplementary R code and data/KM curve/LEF1.survival.tiff]

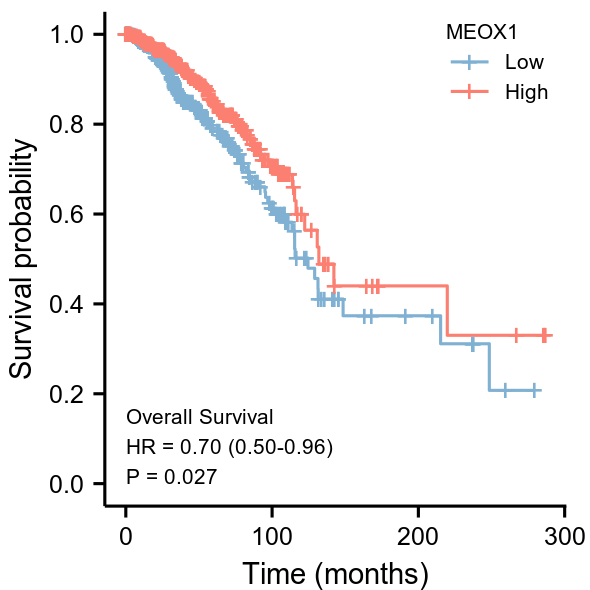

Supplement: Supplementary file 1 — Additional file 1. R code and data. [file 12920_2023_1521_MOESM1_ESM.zip › Supplementary R code and data/KM curve/MEOX1.survival.tiff]

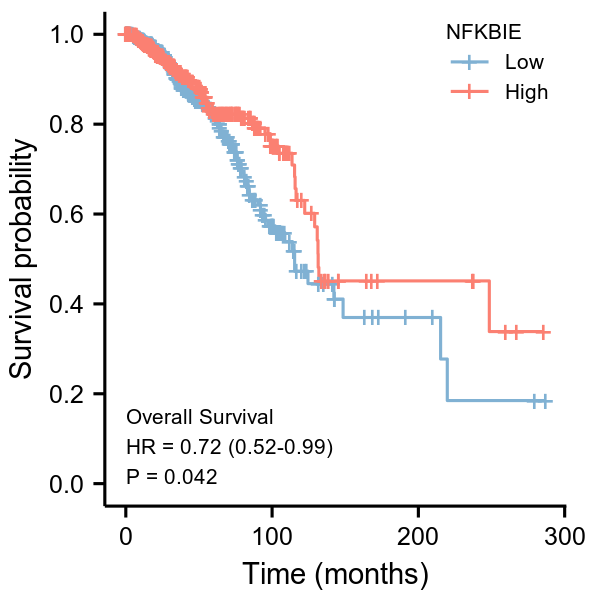

Supplement: Supplementary file 1 — Additional file 1. R code and data. [file 12920_2023_1521_MOESM1_ESM.zip › Supplementary R code and data/KM curve/NFKBIE.survival.tiff]

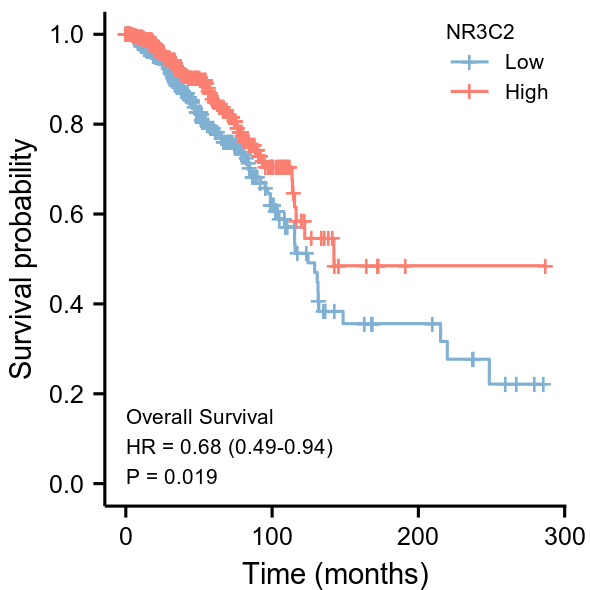

Supplement: Supplementary file 1 — Additional file 1. R code and data. [file 12920_2023_1521_MOESM1_ESM.zip › Supplementary R code and data/KM curve/NR3C2.survival.tiff]

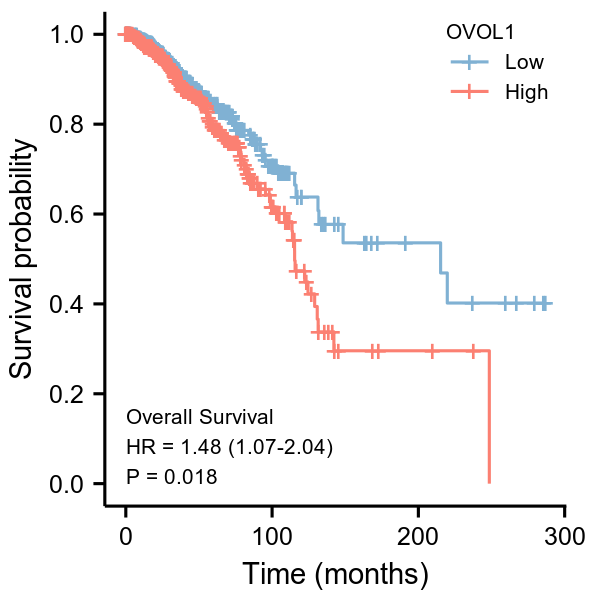

Supplement: Supplementary file 1 — Additional file 1. R code and data. [file 12920_2023_1521_MOESM1_ESM.zip › Supplementary R code and data/KM curve/OVOL1.survival.tiff]

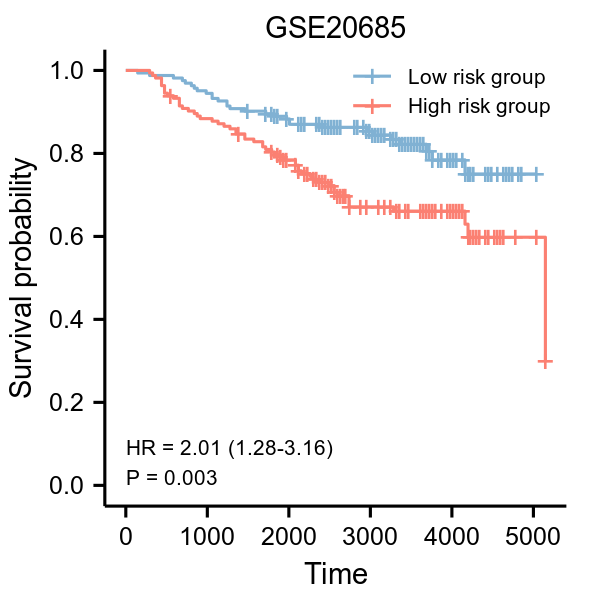

Supplement: Supplementary file 1 — Additional file 1. R code and data. [file 12920_2023_1521_MOESM1_ESM.zip › Supplementary R code and data/KM curve/test.survival.tiff]

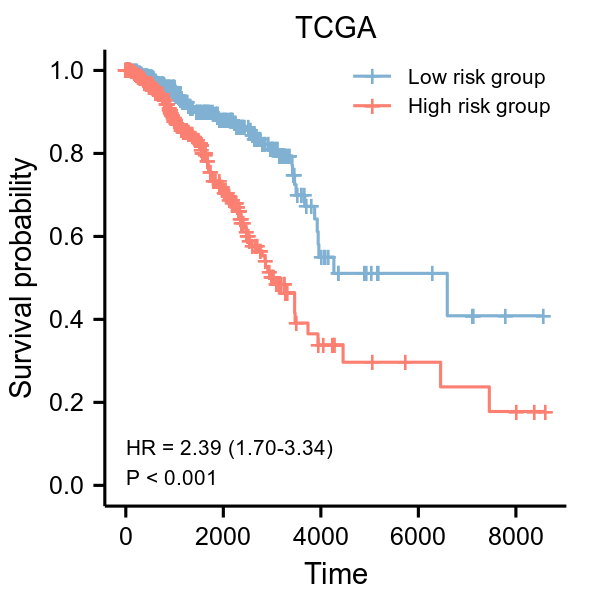

Supplement: Supplementary file 1 — Additional file 1. R code and data. [file 12920_2023_1521_MOESM1_ESM.zip › Supplementary R code and data/KM curve/train.survival.tiff]

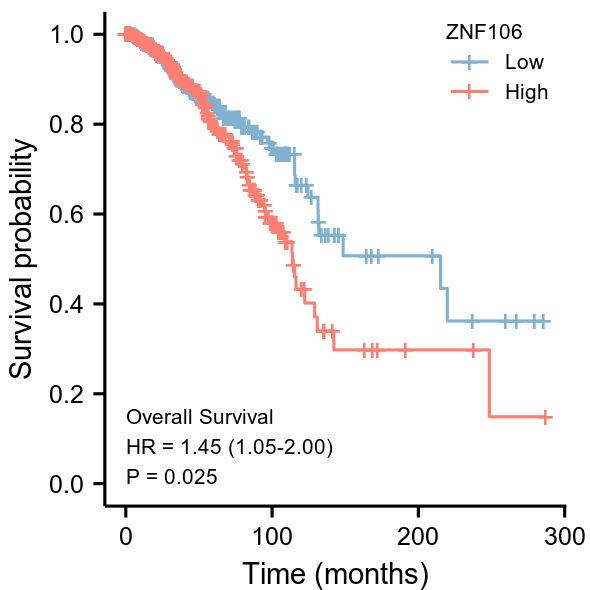

Supplement: Supplementary file 1 — Additional file 1. R code and data. [file 12920_2023_1521_MOESM1_ESM.zip › Supplementary R code and data/KM curve/ZNF106.survival.tiff]

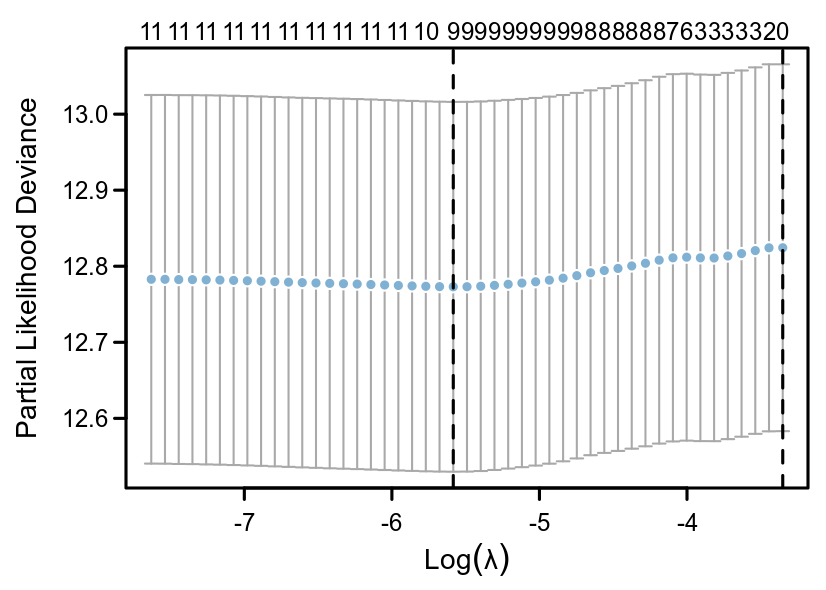

Supplement: Supplementary file 1 — Additional file 1. R code and data. [file 12920_2023_1521_MOESM1_ESM.zip › Supplementary R code and data/LASSO/Lasso.tiff]

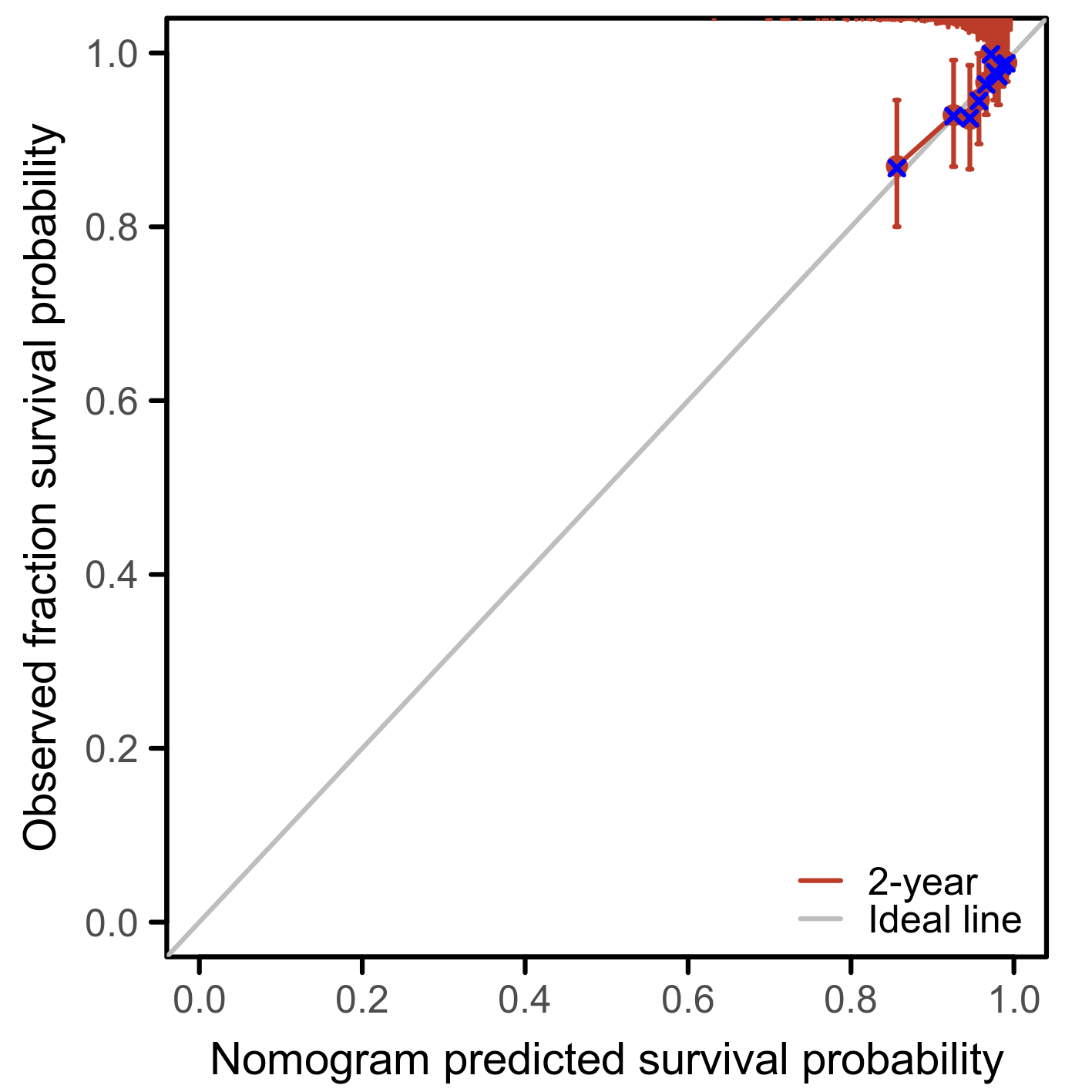

Supplement: Supplementary file 1 — Additional file 1. R code and data. [file 12920_2023_1521_MOESM1_ESM.zip › Supplementary R code and data/Nomogram_calibration/calibration2.tiff]

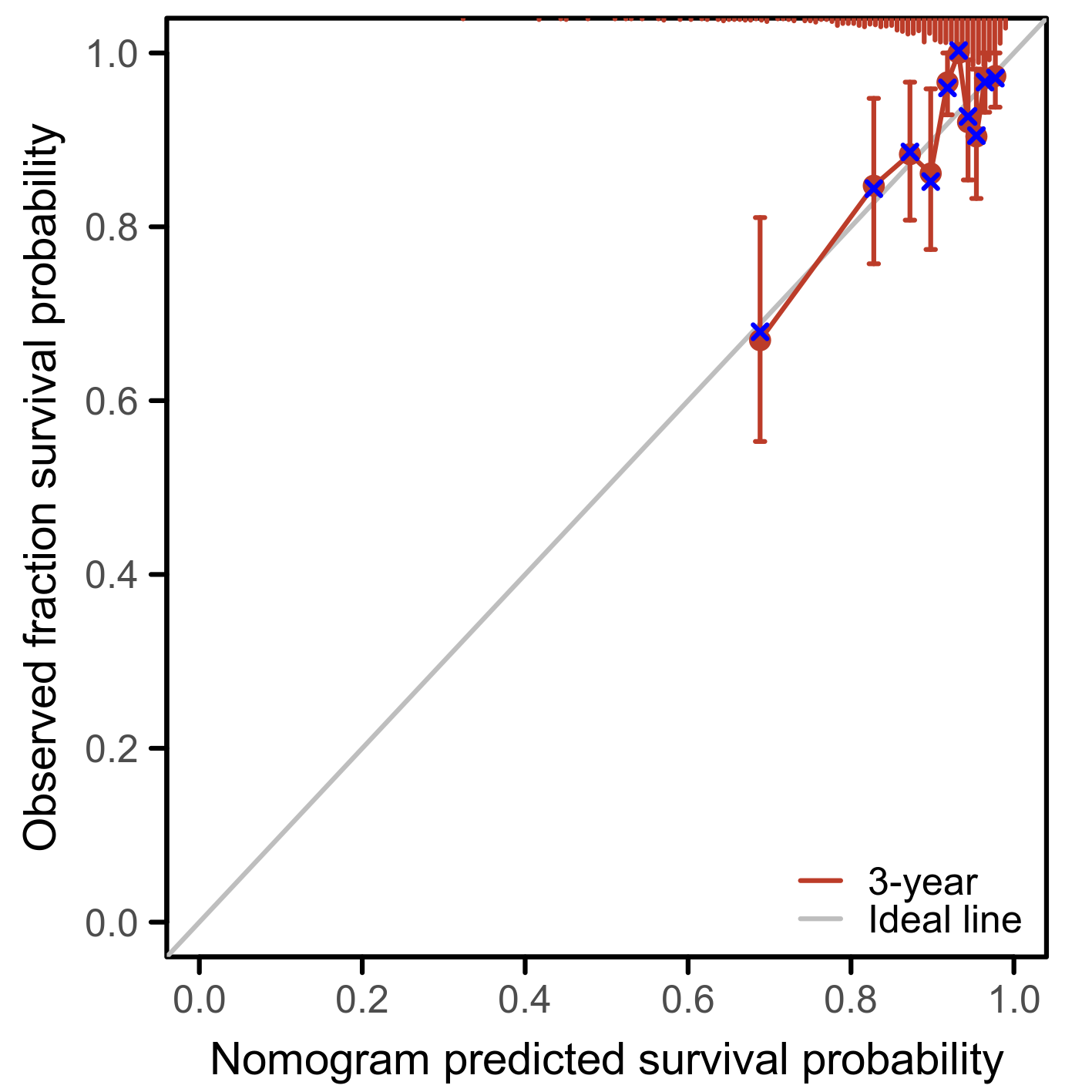

Supplement: Supplementary file 1 — Additional file 1. R code and data. [file 12920_2023_1521_MOESM1_ESM.zip › Supplementary R code and data/Nomogram_calibration/calibration3.tiff]

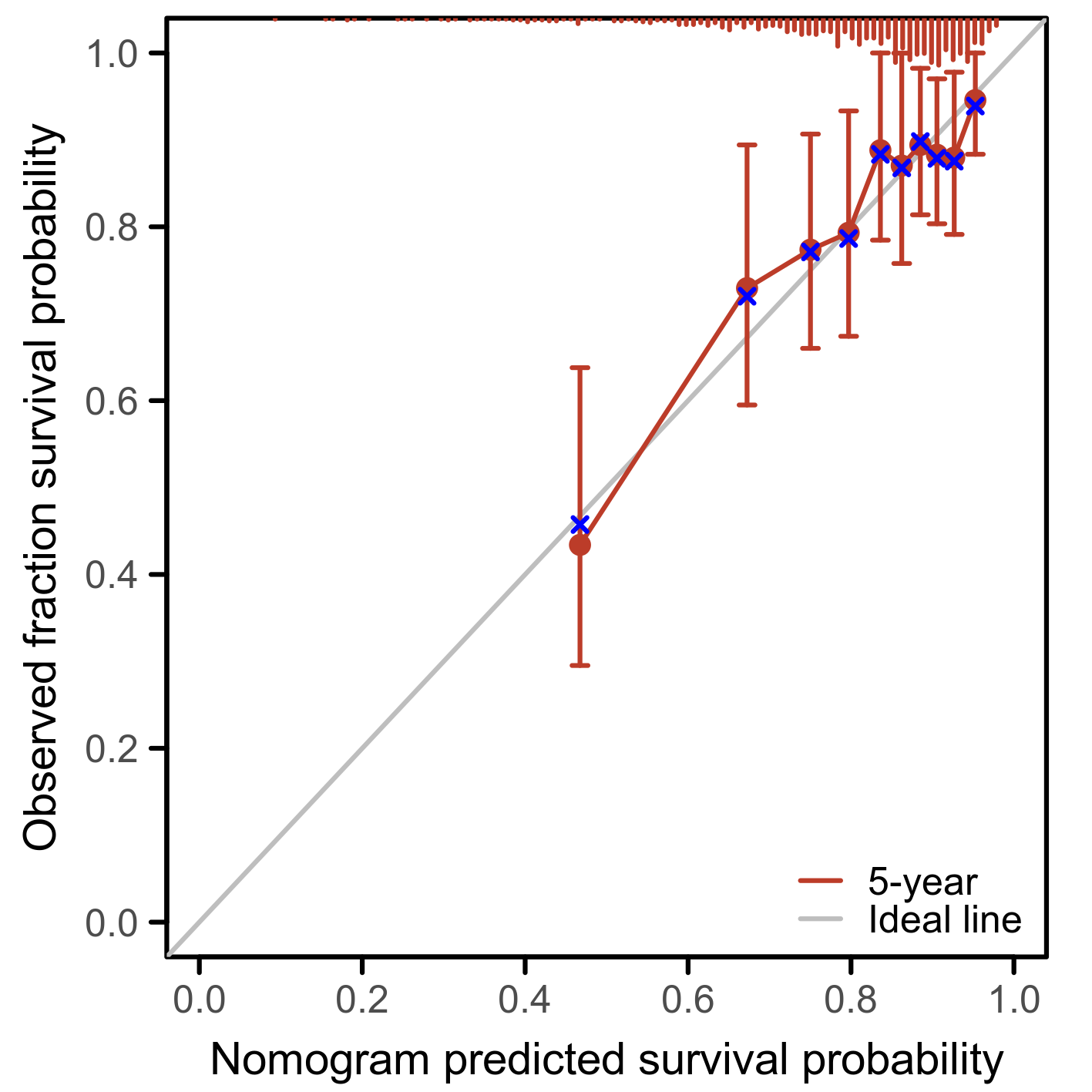

Supplement: Supplementary file 1 — Additional file 1. R code and data. [file 12920_2023_1521_MOESM1_ESM.zip › Supplementary R code and data/Nomogram_calibration/calibration5.tiff]

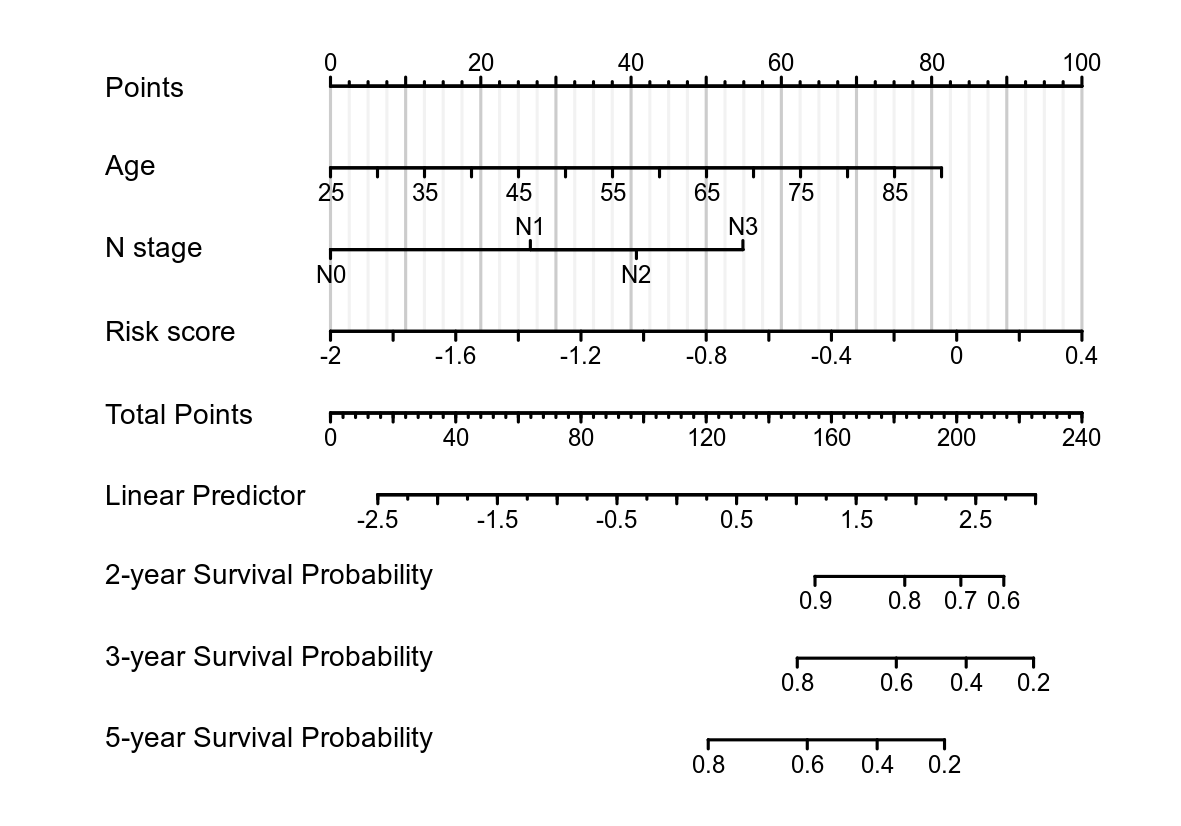

Supplement: Supplementary file 1 — Additional file 1. R code and data. [file 12920_2023_1521_MOESM1_ESM.zip › Supplementary R code and data/Nomogram_calibration/Nomogram.tiff]

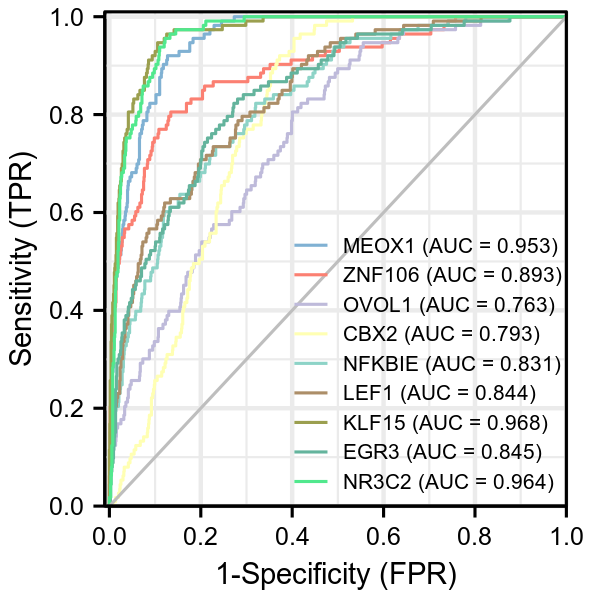

Supplement: Supplementary file 1 — Additional file 1. R code and data. [file 12920_2023_1521_MOESM1_ESM.zip › Supplementary R code and data/ROC/ROC.tiff]

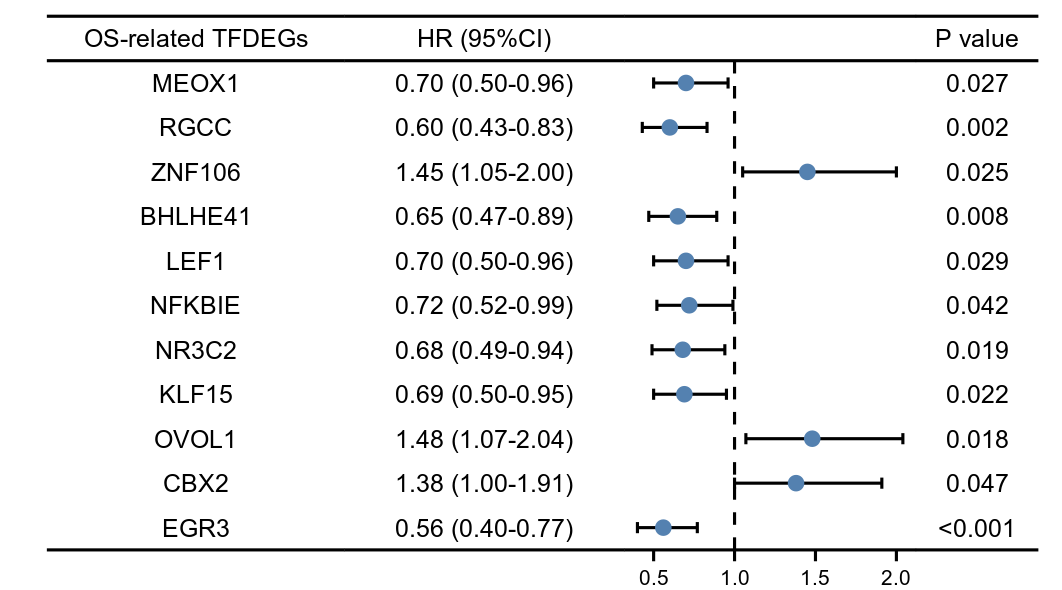

Supplement: Supplementary file 1 — Additional file 1. R code and data. [file 12920_2023_1521_MOESM1_ESM.zip › Supplementary R code and data/univariate Cox/forest.tiff]
