# Supplementary material for: Comprehensive research into prognostic and immune signatures of transcription factor family in breast cancer
Source: BMC Med Genomics. 2023 Apr 25;16:87. doi: 10.1186/s12920-023-01521-y (PMC10127334; doi:10.1186/s12920-023-01521-y)
Supplement: Supplementary file 2 — Additional file 2. Table S1. The TF family genes. [file 12920_2023_1521_MOESM2_ESM.docx]

**Supplementary Table S1. The TF family genes (1536)**

| AATF | FOSB | MED12 | PSMC5 | TPRX1P1 |
| --- | --- | --- | --- | --- |
| ABT1 | FOSL1 | MED14 | PTTG1IP | TPRX2 |
| ACVR2A | FOSL2 | MED15 | PURA | TPRXL |
| ADNP | FOXA1 | MED16 | RARA | TRIM10 |
| ADNP2 | FOXA2 | MED17 | RARB | TRIM13 |
| ADPGK | FOXA3 | MED21 | RARG | TRIM15 |
| AFDN | FOXB1 | MED23 | RASSF7 | TRIM22 |
| AFF1 | FOXC1 | MED24 | RAX | TRIM24 |
| AFF2 | FOXC2 | MED26 | RAX2 | TRIM25 |
| AFF3 | FOXD1 | MED27 | RB1 | TRIM26 |
| AFF4 | FOXD2 | MED6 | RBBP5 | TRIM27 |
| AHR | FOXD3 | MED7 | RBBP9 | TRIM28 |
| AIRE | FOXD4 | MEF2A | RBL1 | TRIM3 |
| AKAP17A | FOXE1 | MEF2C | RBL2 | TRIM33 |
| ALX1 | FOXE3 | MEF2D | RBPJ | TRIM38 |
| ALX3 | FOXF1 | MEFV | RBPJL | TRIM62 |
| ALX4 | FOXF2 | MEIS1 | RCOR1 | TRIOBP |
| ALYREF | FOXG1 | MEIS2 | RCVRN | TRIP10 |
| ANP32A | FOXH1 | MEIS3 | REL | TRIP11 |
| AR | FOXI1 | MEIS3P1 | RELA | TRIP13 |
| ARC | FOXJ1 | MEIS3P2 | RELB | TRIP4 |
| ARFGAP2 | FOXJ2 | MEOX1 | REPIN1 | TRIP6 |
| ARGFX | FOXJ3 | MEOX2 | RERE | TRMT1 |
| ARGFXP1 | FOXK2 | MESP2 | REST | TRPS1 |
| ARGFXP2 | FOXL1 | METTL3 | REV3L | TSC22D1 |
| ARHGAP35 | FOXL2 | MFSD12 | RFX1 | TSC22D2 |
| ARID1B | FOXM1 | MFSD3 | RFX2 | TSC22D3 |
| ARID3A | FOXN1 | MGA | RFX3 | TSC22D4 |
| ARID3B | FOXN2 | MGAT5 | RFX4 | TSHZ1 |
| ARID5A | FOXN3 | MICALL1 | RFX5 | TSHZ2 |
| ARID5B | FOXO1 | MID1 | RFXANK | TSHZ3 |
| ARIH2 | FOXO3 | MIEF1 | RGCC | TTLL4 |
| ARNT | FOXO4 | MIER1 | RHOXF1 | TTLL5 |
| ARNT2 | FOXP1 | MIR9-1HG | RHOXF2 | TUB |
| ARNTL | FOXP2 | MIS18BP1 | RHOXF2B | TULP1 |
| ARX | FOXP3 | MITF | RIMS3 | TULP2 |
| ASCL1 | FOXQ1 | MIXL1 | RING1 | TULP3 |
| ASCL2 | FOXS1 | MKX | RLF | TULP4 |
| ASCL3 | FUBP1 | MLLT1 | RLIM | TWIST1 |
| ASH1L | GABPA | MLLT10 | RNF10 | UBP1 |
| ASH2L | GABPB1 | MLLT3 | RNF103 | UBR4 |
| ATF1 | GATA1 | MLLT6 | RNF112 | UBTF |
| ATF2 | GATA2 | MLX | RNF113A | UHRF1 |
| ATF3 | GATA3 | MLXIP | RNF114 | ULK2 |
| ATF4 | GATA4 | MLXIPL | RNF115 | UNCX |
| ATF5 | GATA5 | MNAT1 | RNF13 | USE1 |
| ATF6 | GATA6 | MNDA | RNF14 | USF1 |
| ATF6B | GBX1 | MNT | RNF141 | USF2 |
| ATF7 | GBX2 | MNX1 | RNF144A | UTF1 |
| ATF7IP | GCM1 | MORF4 | RNF2 | VAX1 |
| ATG4B | GCM2 | MORF4L1 | RNF24 | VAX2 |
| ATN1 | GFI1 | MORF4L2 | RNF4 | VDR |
| ATOH1 | GFI1B | MSC | RNF41 | VENTX |
| ATOH7 | GLI1 | MSRB2 | RNF8 | VENTXP1 |
| ATRX | GLI2 | MSX1 | RORA | VENTXP2 |
| ATXN2 | GLI3 | MSX2 | RORB | VENTXP3 |
| BACH1 | GLIS1 | MSX2P1 | RORC | VENTXP4 |
| BACH2 | GLIS2 | MTA1 | RREB1 | VENTXP5 |
| BANP | GRHL1 | MTA2 | RRN3 | VENTXP6 |
| BARHL1 | GSC | MTF1 | RTRAF | VENTXP7 |
| BARHL2 | GSC2 | MTF2 | RUNX1 | VEZF1 |
| BARX1 | GSX1 | MXD1 | RUNX1T1 | VGLL1 |
| BARX2 | GSX2 | MXD3 | RUNX2 | VPS72 |
| BATF | GTF2A1 | MXD4 | RUNX3 | VSX1 |
| BATF3 | GTF2A1L | MXI1 | RXRA | VSX2 |
| BAZ1A | GTF2A2 | MYB | RXRB | WDR45B |
| BAZ1B | GTF2B | MYBBP1A | RXRG | WT1 |
| BAZ2A | GTF2E1 | MYBL1 | SAFB | XBP1 |
| BAZ2B | GTF2E2 | MYBL2 | SALL1 | YAF2 |
| BCL11A | GTF2F1 | MYC | SALL2 | YBX1 |
| BCL11B | GTF2F2 | MYCBP | SALL3 | YBX2 |
| BCL3 | GTF2H1 | MYCL | SALL4 | YBX3 |
| BCL6 | GTF2H2 | MYCLP1 | SAMD4B | YEATS4 |
| BCLAF1 | GTF2H3 | MYCN | SAP18 | YY1 |
| BHLHE22 | GTF2H4 | MYCNOS | SAP30 | ZAR1 |
| BHLHE40 | GTF2I | MYF5 | SAP30BP | ZBTB11 |
| BHLHE41 | GTF3A | MYF6 | SART3 | ZBTB14 |
| BLOC1S1 | GTF3C1 | MYOD1 | SATB1 | ZBTB16 |
| BLZF1 | GTF3C2 | MYOG | SATB2 | ZBTB17 |
| BMI1 | GTF3C3 | MYRF | SCAND1 | ZBTB18 |
| BNC1 | GTF3C4 | MYT1 | SCAND2P | ZBTB20 |
| BNC2 | GTF3C5 | MYT1L | SCML1 | ZBTB21 |
| BORCS8-MEF2B | GTPBP1 | MZF1 | SCML2 | ZBTB22 |
| BPTF | HAND1 | NAB1 | SCRT1 | ZBTB24 |
| BRD1 | HAND2 | NAB2 | SCRT2 | ZBTB25 |
| BRD2 | HCFC2 | NANOG | SEBOX | ZBTB32 |
| BRD3 | HDAC1 | NANOGP1 | SERTAD1 | ZBTB33 |
| BRD4 | HDAC2 | NANOGP10 | SERTAD2 | ZBTB38 |
| BRD7 | HDAC4 | NANOGP11 | SETBP1 | ZBTB39 |
| BRDT | HDX | NANOGP2 | SETD4 | ZBTB40 |
| BRF1 | HES1 | NANOGP3 | SETDB1 | ZBTB43 |
| BRPF1 | HES2 | NANOGP4 | SF1 | ZBTB47 |
| BRPF3 | HES4 | NANOGP5 | SHOX | ZBTB48 |
| BSX | HES5 | NANOGP6 | SHOX2 | ZBTB6 |
| BTAF1 | HES6 | NANOGP7 | SIAH1 | ZBTB7A |
| BTBD3 | HES7 | NANOGP8 | SIAH2 | ZBTB7B |
| BTF3 | HESX1 | NANOGP9 | SIM1 | ZDHHC1 |
| BTF3P11 | HEXIM1 | NCALD | SIM2 | ZDHHC17 |
| BTF3P12 | HEY1 | NCOA1 | SIN3B | ZEB1 |
| BTF3P13 | HEY2 | NCOA2 | SIX1 | ZEB2 |
| C22orf31 | HEYL | NCOA3 | SIX2 | ZEB2P1 |
| CAND1 | HHEX | NCOA4 | SIX3 | ZFAND3 |
| CASK | HIC1 | NCOA6 | SIX4 | ZFAND5 |
| CASZ1 | HIC2 | NCOR1 | SIX5 | ZFAND6 |
| CBFA2T3 | HIF1A | NCOR2 | SIX6 | ZFHX2 |
| CBX1 | HIF3A | NEUROD1 | SLC25A40 | ZFHX3 |
| CBX2 | HIRA | NEUROD2 | SMAD1 | ZFHX4 |
| CBX3 | HIVEP1 | NEUROD4 | SMAD2 | ZFP28 |
| CBX4 | HIVEP2 | NEUROD6 | SMAD3 | ZFP30 |
| CBX5 | HLF | NEUROG1 | SMAD4 | ZFP36 |
| CBX6 | HLTF | NEUROG2 | SMAD5 | ZFP36L2 |
| CBX7 | HLX | NEUROG3 | SMAD6 | ZFP37 |
| CBX8 | HMBOX1 | NFAT5 | SMAD7 | ZFP62 |
| CCT4 | HMG20B | NFATC1 | SMAD9 | ZFP64 |
| CDK7 | HMGA1 | NFATC2 | SMARCA1 | ZFP69B |
| CDK8 | HMGA2 | NFATC3 | SMARCA2 | ZFP91 |
| CDR2 | HMGB1 | NFATC4 | SMARCA4 | ZFP92 |
| CDX1 | HMGB2 | NFE2 | SMARCA5 | ZFPL1 |
| CDX2 | HMGN2 | NFE2L1 | SMARCAD1 | ZFPM1 |
| CDX4 | HMGXB3 | NFE2L2 | SMARCAL1 | ZFPM2 |
| CEBPA | HMX1 | NFE2L3 | SMARCB1 | ZFR |
| CEBPB | HMX2 | NFIA | SMARCC1 | ZFX |
| CEBPD | HMX3 | NFIB | SMARCC2 | ZFY |
| CEBPE | HNF1A | NFIC | SMARCE1 | ZHX1 |
| CEBPG | HNF1B | NFIL3 | SMYD5 | ZHX2 |
| CEBPZ | HNF4A | NFIX | SNAI1 | ZHX3 |
| CELF3 | HNF4G | NFKB1 | SNAI2 | ZIC1 |
| CERS2 | HOMEZ | NFKB2 | SNAI3 | ZIC2 |
| CERS3 | HOPX | NFKBIA | SNAPC1 | ZIC3 |
| CERS4 | HOXA1 | NFKBIB | SNAPC2 | ZIC4 |
| CERS5 | HOXA10 | NFKBIE | SNAPC3 | ZIC5 |
| CERS6 | HOXA11 | NFKBIL1 | SNAPC4 | ZIM2 |
| CFAP20 | HOXA13 | NFRKB | SNAPC5 | ZKSCAN1 |
| CHD1 | HOXA2 | NFX1 | SND1 | ZKSCAN3 |
| CHD1L | HOXA3 | NFYA | SNW1 | ZKSCAN4 |
| CHD2 | HOXA4 | NFYB | SOX1 | ZKSCAN5 |
| CHD3 | HOXA5 | NFYC | SOX10 | ZKSCAN7 |
| CHD4 | HOXA6 | NHLH2 | SOX11 | ZKSCAN8 |
| CHD5 | HOXA7 | NKRF | SOX12 | ZMYM3 |
| CHD6 | HOXA9 | NKX1-1 | SOX13 | ZMYM4 |
| CHMP3 | HOXB1 | NKX1-2 | SOX14 | ZMYM5 |
| CIAO1 | HOXB13 | NKX2-1 | SOX15 | ZMYM6 |
| CIC | HOXB2 | NKX2-2 | SOX17 | ZMYND11 |
| CIITA | HOXB3 | NKX2-3 | SOX18 | ZNF10 |
| CIR1 | HOXB4 | NKX2-4 | SOX2 | ZNF106 |
| CITED1 | HOXB5 | NKX2-5 | SOX21 | ZNF107 |
| CITED2 | HOXB6 | NKX2-6 | SOX3 | ZNF112 |
| CLOCK | HOXB7 | NKX2-8 | SOX30 | ZNF117 |
| CNBP | HOXB8 | NKX3-1 | SOX4 | ZNF12 |
| CNOT3 | HOXB9 | NKX3-2 | SOX5 | ZNF123P |
| CNOT4 | HOXC10 | NKX6-1 | SOX6 | ZNF124 |
| CNOT8 | HOXC11 | NKX6-2 | SOX8 | ZNF131 |
| CNPY3 | HOXC12 | NKX6-3 | SOX9 | ZNF132 |
| COPS2 | HOXC13 | NMI | SP1 | ZNF133 |
| COPS5 | HOXC4 | NOBOX | SP2 | ZNF134 |
| CORO1A | HOXC5 | NOTO | SP3 | ZNF135 |
| CREB1 | HOXC6 | NPAS1 | SP4 | ZNF136 |
| CREB3 | HOXC8 | NPAS2 | SPDEF | ZNF137P |
| CREB3L1 | HOXC9 | NR0B1 | SPEN | ZNF138 |
| CREB5 | HOXD1 | NR0B2 | SPI1 | ZNF14 |
| CREBBP | HOXD10 | NR1D1 | SPIB | ZNF140 |
| CREBL2 | HOXD11 | NR1D2 | SRA1 | ZNF141 |
| CREBZF | HOXD12 | NR1H2 | SRCAP | ZNF142 |
| CREG1 | HOXD13 | NR1H3 | SREBF1 | ZNF143 |
| CREM | HOXD3 | NR1H4 | SREBF2 | ZNF146 |
| CRIP1 | HOXD4 | NR1I2 | SRF | ZNF148 |
| CRIP2 | HOXD8 | NR1I3 | SRY | ZNF154 |
| CRX | HOXD9 | NR2C1 | SSRP1 | ZNF155 |
| CSRP1 | HR | NR2C2 | SSX1 | ZNF157 |
| CSRP2 | HSF1 | NR2E1 | SSX2 | ZNF16 |
| CSRP3 | HSF2 | NR2E3 | SSX3 | ZNF160 |
| CTCF | HSF2BP | NR2F1 | SSX4 | ZNF165 |
| CTNNB1 | HSF4 | NR2F2 | SSX5 | ZNF169 |
| CUX1 | HSFX1 | NR2F6 | SSX6P | ZNF17 |
| CUX2 | HSFY1 | NR3C1 | SSX7 | ZNF174 |
| CUX2P1 | ID1 | NR3C2 | SSX8P | ZNF175 |
| DACH1 | ID2 | NR4A1 | SSX9P | ZNF177 |
| DBP | ID3 | NR4A2 | ST18 | ZNF18 |
| DBX1 | ID4 | NR4A3 | STAT1 | ZNF180 |
| DBX2 | IER2 | NR5A1 | STAT2 | ZNF182 |
| DCP1A | IFT172 | NR5A2 | STAT3 | ZNF184 |
| DDIT3 | IGHMBP2 | NR6A1 | STAT4 | ZNF185 |
| DEAF1 | IKZF1 | NRF1 | STAT5A | ZNF189 |
| DENND4A | IKZF2 | NRIP1 | STAT5B | ZNF19 |
| DEPDC7 | IKZF3 | NRL | STAT6 | ZNF195 |
| DIDO1 | IKZF5 | NSD2 | STON1 | ZNF197 |
| DLX1 | ILF2 | NUFIP2 | SUB1 | ZNF2 |
| DLX2 | ILF3 | OLIG1 | SUPT16H | ZNF20 |
| DLX3 | INSM1 | OLIG2 | SUPT20H | ZNF200 |
| DLX4 | INSM2 | OLIG3 | SUPT3H | ZNF202 |
| DLX5 | INTS4 | ONECUT1 | SUPT4H1 | ZNF205 |
| DLX6 | IRF1 | ONECUT2 | SUPT5H | ZNF207 |
| DMBX1 | IRF2 | ONECUT3 | SUPT6H | ZNF208 |
| DMRT1 | IRF3 | OPTN | TADA2A | ZNF211 |
| DMRT2 | IRF4 | OSR1 | TADA3 | ZNF212 |
| DMRTA2 | IRF5 | OTP | TAF1 | ZNF213 |
| DMRTC1 | IRF6 | OTUD7B | TAF10 | ZNF214 |
| DMTF1 | IRF7 | OTX1 | TAF11 | ZNF215 |
| DPF1 | IRF8 | OTX2 | TAF12 | ZNF217 |
| DPF2 | IRF9 | OTX2P1 | TAF13 | ZNF219 |
| DPF3 | IRX1 | OVOL1 | TAF15 | ZNF22 |
| DPRX | IRX1P1 | OVOL3 | TAF1A | ZNF221 |
| DPRXP1 | IRX2 | PACS2 | TAF1B | ZNF222 |
| DPRXP2 | IRX3 | PATZ1 | TAF1C | ZNF223 |
| DPRXP3 | IRX4 | PAX1 | TAF2 | ZNF224 |
| DPRXP4 | IRX5 | PAX2 | TAF3 | ZNF225 |
| DPRXP5 | IRX6 | PAX3 | TAF4 | ZNF226 |
| DPRXP6 | ISL1 | PAX4 | TAF4B | ZNF229 |
| DPRXP7 | ISL2 | PAX5 | TAF5 | ZNF23 |
| DR1 | ISX | PAX6 | TAF5L | ZNF230 |
| DRAP1 | JARID2 | PAX7 | TAF6 | ZNF232 |
| DRGX | JMJD4 | PAX8 | TAF6L | ZNF234 |
| DTX2 | JUN | PAX9 | TAF7 | ZNF235 |
| DUX4 | JUNB | PAXIP1 | TAF9 | ZNF236 |
| DUX4L8 | JUND | PBX1 | TAL1 | ZNF239 |
| DUXA | KAT2A | PBX2 | TAL2 | ZNF24 |
| DUXAP1 | KAT5 | PBX2P1 | TBC1D10B | ZNF248 |
| DUXAP10 | KAT6A | PBX3 | TBL1Y | ZNF25 |
| DUXAP2 | KAT6B | PBX4 | TBP | ZNF250 |
| DUXAP3 | KAT7 | PCGF2 | TBPL1 | ZNF251 |
| DUXAP4 | KCNIP3 | PCGF3 | TBR1 | ZNF254 |
| DUXAP5 | KDM3B | PDLIM5 | TBX1 | ZNF256 |
| DUXAP6 | KDM4C | PDX1 | TBX10 | ZNF26 |
| DUXAP7 | KDM4D | PER1 | TBX15 | ZNF263 |
| DUXAP8 | KDM5C | PER2 | TBX18 | ZNF264 |
| DUXAP9 | KEAP1 | PER3 | TBX19 | ZNF266 |
| DUXB | KIAA0040 | PFDN5 | TBX2 | ZNF267 |
| E2F1 | KLF1 | PGR | TBX20 | ZNF268 |
| E2F2 | KLF10 | PHC1 | TBX21 | ZNF271P |
| E2F3 | KLF11 | PHF11 | TBX22 | ZNF273 |
| E2F4 | KLF12 | PHF3 | TBX3 | ZNF274 |
| E2F5 | KLF13 | PHOX2A | TBX4 | ZNF275 |
| E2F6 | KLF14 | PHOX2B | TBX5 | ZNF277 |
| E4F1 | KLF15 | PHRF1 | TBX6 | ZNF28 |
| EBF1 | KLF16 | PHTF1 | TBXT | ZNF281 |
| EBF4 | KLF2 | PIAS2 | TCEAL1 | ZNF282 |
| ECD | KLF3 | PIAS3 | TCEAL9 | ZNF286A |
| EED | KLF4 | PITX1 | TCERG1 | ZNF287 |
| EGLN2 | KLF5 | PITX2 | TCF12 | ZNF29P |
| EGR1 | KLF6 | PITX3 | TCF15 | ZNF3 |
| EGR2 | KLF7 | PKNOX1 | TCF19 | ZNF30 |
| EGR3 | KLF8 | PKNOX2 | TCF20 | ZNF304 |
| EGR4 | KLF9 | PLAG1 | TCF21 | ZNF318 |
| EHF | KLHL21 | PLAGL1 | TCF3 | ZNF319 |
| ELF1 | KLHL5 | PLAGL2 | TCF4 | ZNF32 |
| ELF2 | KMT2A | PLRG1 | TCF7 | ZNF324 |
| ELF3 | KMT2D | PMF1 | TCF7L1 | ZNF331 |
| ELF4 | KMT5B | PML | TCF7L2 | ZNF334 |
| ELF5 | L3MBTL1 | POU1F1 | TCFL5 | ZNF337 |
| ELK1 | LARP1 | POU2AF1 | TEAD1 | ZNF33A |
| ELK3 | LAS1L | POU2F1 | TEAD2 | ZNF33B |
| ELK4 | LBX1 | POU2F2 | TEAD3 | ZNF35 |
| EMX1 | LBX2 | POU2F3 | TEAD4 | ZNF354A |
| EMX2 | LDB1 | POU3F1 | TECPR1 | ZNF358 |
| EN1 | LDB2 | POU3F2 | TEF | ZNF362 |
| EN2 | LDOC1 | POU3F3 | TFAM | ZNF37A |
| ENO1 | LEF1 | POU3F4 | TFAP2A | ZNF395 |
| EOMES | LEUTX | POU4F1 | TFAP2B | ZNF408 |
| EP300 | LHX1 | POU4F2 | TFAP2C | ZNF41 |
| EP400 | LHX2 | POU4F3 | TFAP2D | ZNF410 |
| EPAS1 | LHX3 | POU5F1 | TFAP4 | ZNF414 |
| ERCC2 | LHX4 | POU5F1B | TFB1M | ZNF415 |
| ERCC3 | LHX5 | POU5F1P2 | TFB2M | ZNF416 |
| ERCC6 | LHX6 | POU5F1P3 | TFCP2 | ZNF423 |
| ERF | LHX8 | POU5F1P4 | TFCP2L1 | ZNF43 |
| ERG | LHX9 | POU5F1P5 | TFDP1 | ZNF430 |
| ERGIC2 | LIMA1 | POU5F1P6 | TFDP2 | ZNF432 |
| ERMP1 | LIN28A | POU5F1P7 | TFDP3 | ZNF44 |
| ESR1 | LITAF | POU5F2 | TFE3 | ZNF443 |
| ESR2 | LMO1 | POU6F1 | TFEB | ZNF444 |
| ESRRA | LMO2 | POU6F2 | TFEC | ZNF45 |
| ESRRB | LMO3 | PPARA | TGFB1I1 | ZNF460 |
| ESRRG | LMO4 | PPARD | TGIF1 | ZNF461 |
| ESX1 | LMO7 | PPARG | TGIF1P1 | ZNF473 |
| ETS1 | LMX1A | PPARGC1A | TGIF2 | ZNF483 |
| ETS2 | LMX1B | PPP1R13L | TGIF2LX | ZNF510 |
| ETV1 | LRRC14 | PRDM1 | TGIF2LY | ZNF516 |
| ETV2 | LSR | PRDM10 | TGIF2P1 | ZNF532 |
| ETV3 | LTN1 | PRDM11 | THRA | ZNF544 |
| ETV4 | LYL1 | PRDM12 | THRAP3 | ZNF552 |
| ETV5 | LZTR1 | PRDM13 | THRB | ZNF559 |
| ETV6 | LZTS1 | PRDM14 | TICRR | ZNF576 |
| ETV7 | MAF | PRDM15 | TIMELESS | ZNF581 |
| EVX1 | MAFB | PRDM16 | TLX1 | ZNF592 |
| EVX2 | MAFF | PRDM2 | TLX2 | ZNF593 |
| EYA1 | MAFG | PRDM4 | TLX3 | ZNF605 |
| EYA2 | MAFK | PRDM5 | TMEM121B | ZNF613 |
| EYA3 | MAML3 | PRDM6 | TMEM259 | ZNF629 |
| EYA4 | MAMLD1 | PRDM7 | TMEM260 | ZNF630 |
| EZH1 | MANSC1 | PRDM8 | TMF1 | ZNF639 |
| EZH2 | MAPK8IP1 | PRDM9 | TNRC18P2 | ZNF646 |
| FAM189B | MAX | PREB | TNRC6A | ZNF649 |
| FAM20C | MAZ | PRICKLE3 | TONSL | ZNF668 |
| FEM1A | MBD1 | PROP1 | TOX | ZNF669 |
| FEZF2 | MBD2 | PROX1 | TOX3 | ZNF672 |
| FHL1 | MBD4 | PROX2 | TP53 | ZNF682 |
| FHL2 | MBNL2 | PRR7 | TP53I13 | ZNF691 |
| FHL5 | MECOM | PRRX1 | TP63 | ZNF692 |
| FLI1 | MECP2 | PRRX2 | TP73 | ZNF696 |
| FOS | MED1 | PSIP1 | TPRX1 | ZNF7 |
| ZNF701 | ZNF80 | ZNF92 | ZSCAN22 | ZSCAN12 |
| ZNF706 | ZNF81 | ZNF93 | ZSCAN26 | ZSCAN2 |
| ZNF708 | ZNF823 | ZNHIT6 | ZSCAN9 | ZSCAN20 |
| ZNF711 | ZNF83 | ZPR1 | ZXDA | ZSCAN21 |
| ZNF74 | ZNF831 | ZRANB2 | ZXDB | ZNF90 |
| ZNF746 | ZNF84 | ZSCAN10 | ZXDC | ZNF91 |
| ZNF75A | ZNF85 | ZNF79 | ZNF875 | ZNF8 |
| ZNF76 |  |  |  |  |
