# Supplementary material for: Comprehensive research into prognostic and immune signatures of transcription factor family in breast cancer
Source: BMC Med Genomics. 2023 Apr 25;16:87. doi: 10.1186/s12920-023-01521-y (PMC10127334; doi:10.1186/s12920-023-01521-y)
Supplement: Supplementary file 3 — Additional file 3: Figure S1. Kaplan–Meier curves for OS prediction in TCGA-BRCA of A MEOX1; B RGCC; C ZNF106; D BHLHE41; E LEF1; F NFKBIE; G NR3C2; H KLF15; I OVOL1; J CBX2; K EGR3. [file 12920_2023_1521_MOESM3_ESM.docx]

**
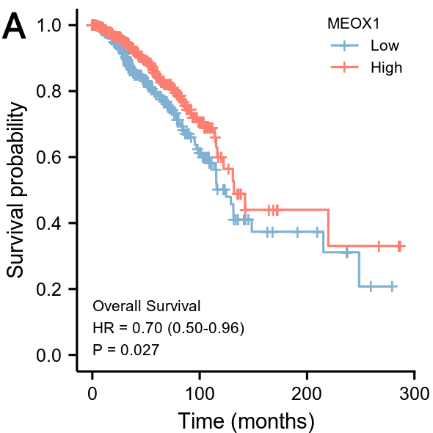

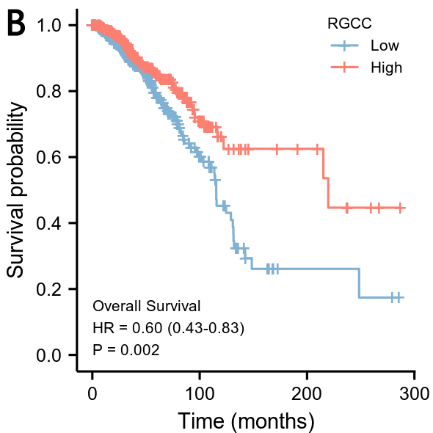

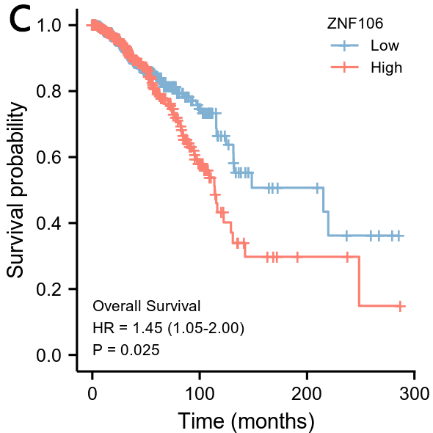

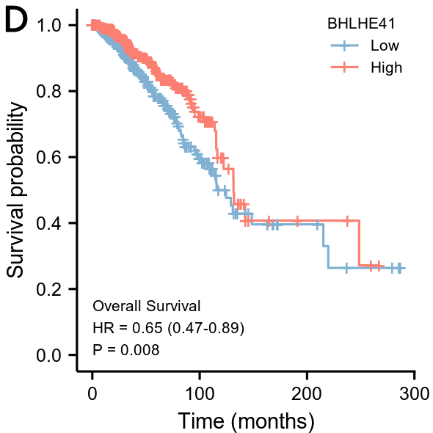

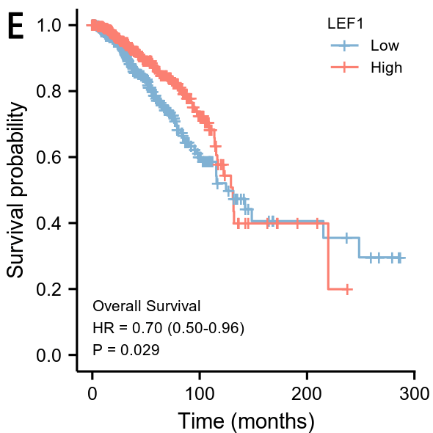

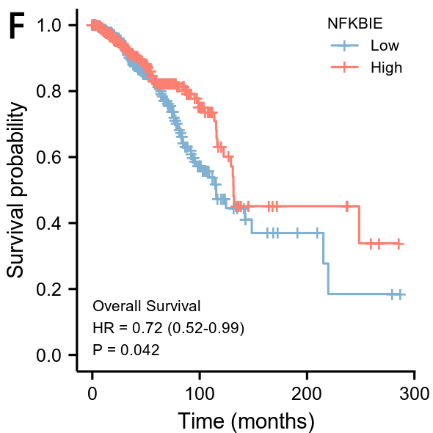

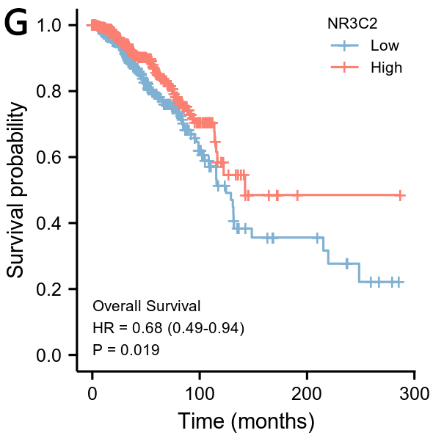

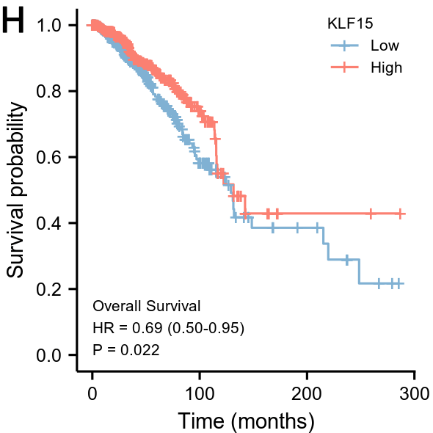

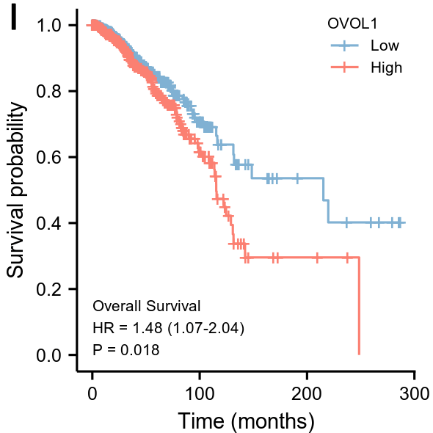

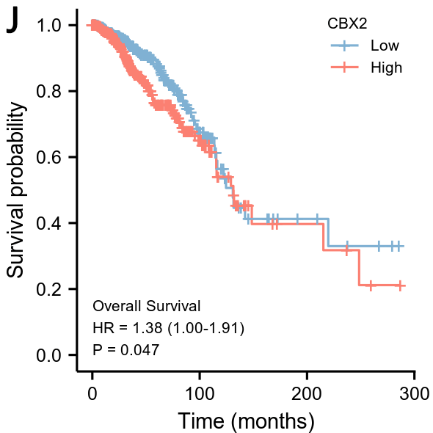

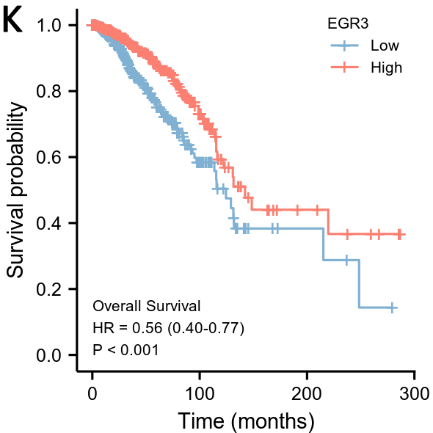
**

**Supplementary Figure S1.** Kaplan–Meier curves for OS prediction in TCGA-BRCA of (A) MEOX1; (B) RGCC; (C) ZNF106; (D) BHLHE41; (E) LEF1; (F) NFKBIE; (G) NR3C2; (H) KLF15; (I) OVOL1; (J) CBX2; (K) EGR3.
