# Supplementary material for: Comprehensive research into prognostic and immune signatures of transcription factor family in breast cancer
Source: BMC Med Genomics. 2023 Apr 25;16:87. doi: 10.1186/s12920-023-01521-y (PMC10127334; doi:10.1186/s12920-023-01521-y)
Supplement: Supplementary file 4 — Additional file 4: Figure S2. Kaplan–Meier curves for OS prediction in BRCA subtypes of A Her2 or Basal and B M1. [file 12920_2023_1521_MOESM4_ESM.docx]

**
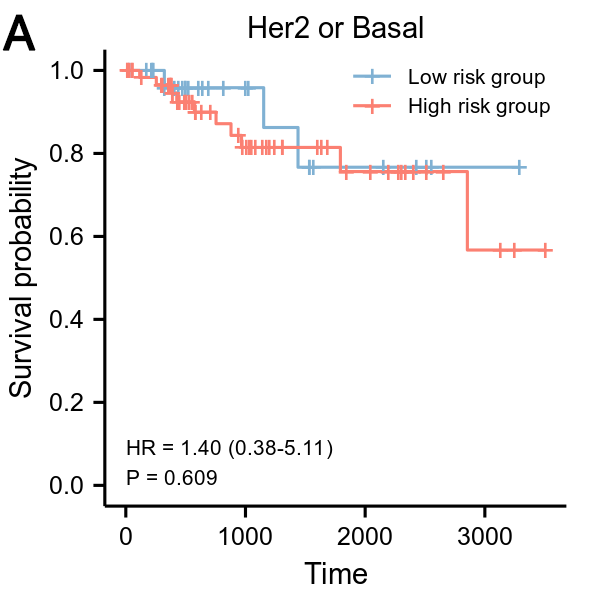

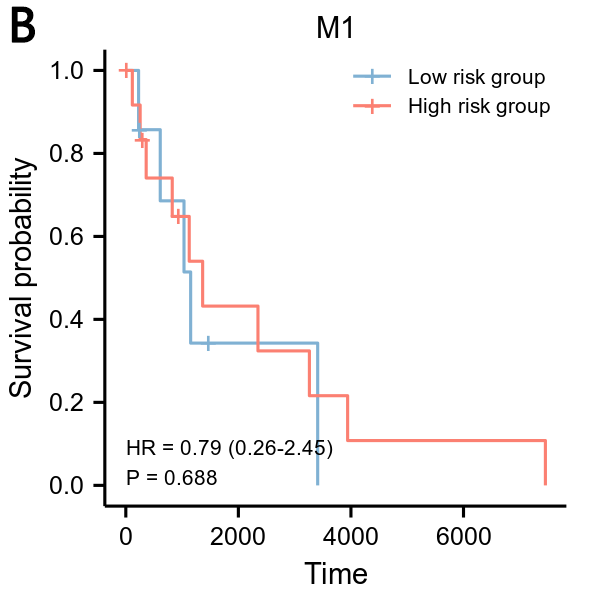
**

**Supplementary Figure S2.** Kaplan–Meier curves for OS prediction in BRCA subtypes of (A) Her2 or Basal and (B) M1.
