# Supplementary material for: Comprehensive research into prognostic and immune signatures of transcription factor family in breast cancer
Source: BMC Med Genomics. 2023 Apr 25;16:87. doi: 10.1186/s12920-023-01521-y (PMC10127334; doi:10.1186/s12920-023-01521-y)
Supplement: Supplementary file 5 — Additional file 5: Figure S3. Heatmap of DNA methylation expression levels of the TFDEGs in BRCA by MethSurv platform. [file 12920_2023_1521_MOESM5_ESM.docx]

**
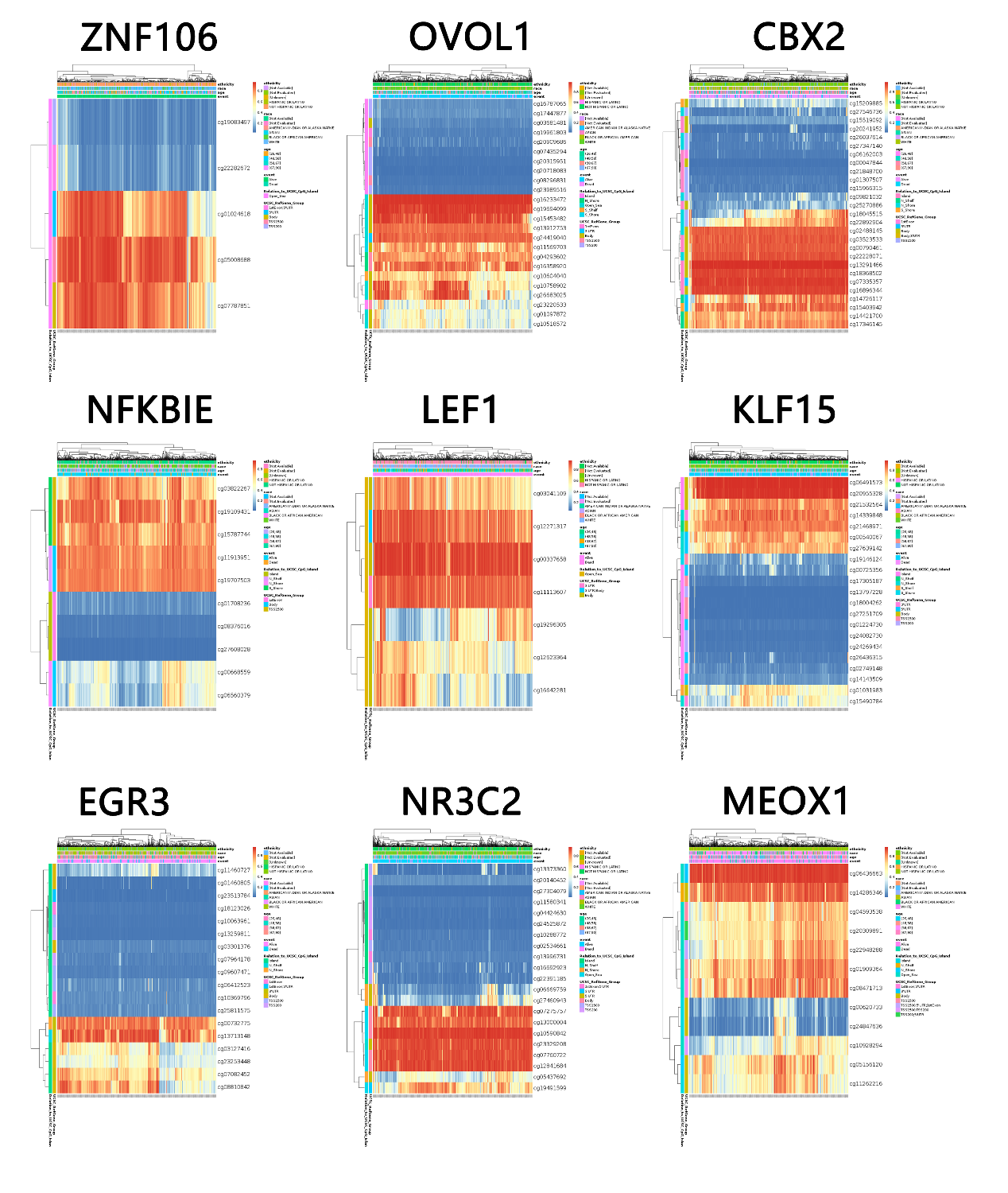
Supplementary Figure S3. Heatmap of DNA methylation expression levels of the TFDEGs in BRCA by MethSurv platform.**

cg01024618, cg05008688, cg07787851 of ZNF106; cg16233472, cg19694099, cg15453482 of OVOL1; cg02488145, cg22228071, cg18045515, cg22892904, cg00790461, cg03523533, cg13291466, cg16896344, cg18368502, cg07335357, cg14421700, cg14726117, cg17346145, cg15403942 of CBX2; cg03822267, cg19109431, cg15787744, cg11913951, cg19707503 of NFKBIE; cg12271317, cg00337658, cg11113607 of LEF1; cg06491573, cg20955328 of KLF15; cg00732775, cg13713148, cg03127416, cg23253448, cg07082452, cg08810842 of EGR3; cg07275757, cg13000004, cg10590842, cg23329208, cg07760722, cg12841684 of NR3C2; cg06436663, cg14286346 of MEOX1 displays the highest level of DNA methylation in BRCA.
