# Supplementary material for: Comprehensive research into prognostic and immune signatures of transcription factor family in breast cancer
Source: BMC Med Genomics. 2023 Apr 25;16:87. doi: 10.1186/s12920-023-01521-y (PMC10127334; doi:10.1186/s12920-023-01521-y)
Supplement: Supplementary file 6 — Additional file 6: Table S2. Prognostic Value of Single CpG of the TFDEGs in BRCA by MethSurv platform. [file 12920_2023_1521_MOESM6_ESM.docx]

**Supplementary Table S2: Prognostic Value of Single CpG of the TFDEGs in BRCA by MethSurv platform.**

| **Gene-CpG** | **HR** | **LR Test p-value** |
| --- | --- | --- |
| **ZNF106-5'UTR-Open_Sea-cg01024618** | **1.641** | **0.019*** |
| **ZNF106-TSS500-Open_Sea-cg05008688** | **1.554** | **0.038*** |
| **ZNF106-Body-Open_Sea-cg07787851** | **1.593** | **0.041*** |
| **OVOL1-Body-Open_Sea-cg16233472** | **0.58** | **0.0063*** |
| **OVOL1-Body-Open_Sea-cg19694099** | **0.644** | **0.028*** |
| **OVOL1-Body-Open_Sea-cg15453482** | **0.466** | **0.00017*** |
| CBX2-Body-N_Shore-cg02488145 | 1.254 | 0.38 |
| **CBX2-Body-N_Shore-cg22228071** | **2.136** | **0.0068*** |
| **CBX2-TSS1500-N_Shore-cg18045515** | **0.544** | **0.0051*** |
| **CBX2-TSS1500-N_Shore-cg22892904** | **0.455** | **0.000095*** |
| CBX2-Body-Island-cg00790461 | 1.307 | 0.29 |
| CBX2-Body-Island-cg03523533 | 0.767 | 0.24 |
| CBX2-Body-Island-cg13291466 | 0.798 | 0.34 |
| CBX2-Body-Island-cg16896344 | 1.383 | 0.1 |
| CBX2-Body-Island-cg18368502 | 0.722 | 0.15 |
| **CBX2-3'UTR-Island-cg07335357** | **1.772** | **0.018*** |
| CBX2-Body;3'UTR-N_Shelf-cg14421700 | 0.686 | 0.058 |
| **CBX2-3'UTR-N_Shelf-cg14726117** | **0.543** | **0.017*** |
| **CBX2-Body-N_Shelf-cg17346145** | **0.633** | **0.026*** |
| CBX2-3'UTR-S_Shore-cg15403942 | 0.764 | 0.23 |
| NFKBIE-TSS1500-S_Shore-cg03822267 | 0.757 | 0.17 |
| **NFKBIE-TSS1500-S_Shore-cg19109431** | **0.593** | **0.031*** |
| NFKBIE-TSS1500-S_Shore-cg15787744 | 1.288 | 0.32 |
| NFKBIE-Body-N-Shelf-cg11913951 | 1.424 | 0.15 |
| NFKBIE-Body-N-Shelf-cg19707503 | 0.815 | 0.31 |
| **LEF1-3'UTR; Body-Open_Sea-cg12271317** | **0.485** | **0.00029*** |
| **LEF1-Body-Open_Sea-cg00337658** | **0.476** | **0.00018*** |
| **LEF1-3'UTR-Open_Sea-cg11113607** | **0.525** | **0.014*** |
| KLF15-Body-Island-cg06491573 | 1.525 | 0.05 |
| KLF15-Body-Island-cg20955328 | 1.336 | 0.21 |
| EGR3-3'UTR-N_Shore-cg00732775 | 1.153 | 0.49 |
| EGR3-3'UTR-N_Shelf-cg13713148 | 1.506 | 0.063 |
| EGR3-Body-Island-cg03127416 | 0.782 | 0.29 |
| EGR3-Body-Island-cg23253448 | 1.365 | 0.12 |
| EGR3-3'UTR-Island-cg07082452 | 1.169 | 0.51 |
| EGR3-Body-Island-cg08810842 | 1.449 | 0.09 |
| **NR3C2-Body-Open_Sea-cg07275757** | **0.52** | **0.0098*** |
| NR3C2-Body-Open_Sea-cg13000004 | 1.311 | 0.18 |
| **NR3C2-Body-Open_Sea-cg10590842** | **0.624** | **0.027*** |
| NR3C2-5'UTR-N-Shelf-cg23329208 | 1.278 | 0.26 |
| NR3C2-Body-Open_Sea-cg07760722 | 0.717 | 0.11 |
| NR3C2-Body-Open_Sea-cg12841684 | 1.312 | 0.26 |
| MEOX1-3'UTR-Open_Sea-cg06436663 | 0.662 | 0.08 |
| MEOX1-Body-N_Shelf-cg14286346 | 1.471 | 0.054 |

The threshold of significance was LR Test p-value <0.05.
