# Supplementary material for: Comprehensive research into prognostic and immune signatures of transcription factor family in breast cancer
Source: BMC Med Genomics. 2023 Apr 25;16:87. doi: 10.1186/s12920-023-01521-y (PMC10127334; doi:10.1186/s12920-023-01521-y)
Supplement: Supplementary file 7 — Additional file 7: Figure S4. Correlations between expressions of TFDEGs and immune infiltration profiles in BRCA. The figure shows the expression of each gene associated with tumor purity and several tumor-infiltrating immune cell markers, such as B cell, CD8+ T cell, CD4 + T cell, macrophage, neutrophil, and dendritic cell markers. [file 12920_2023_1521_MOESM7_ESM.docx]

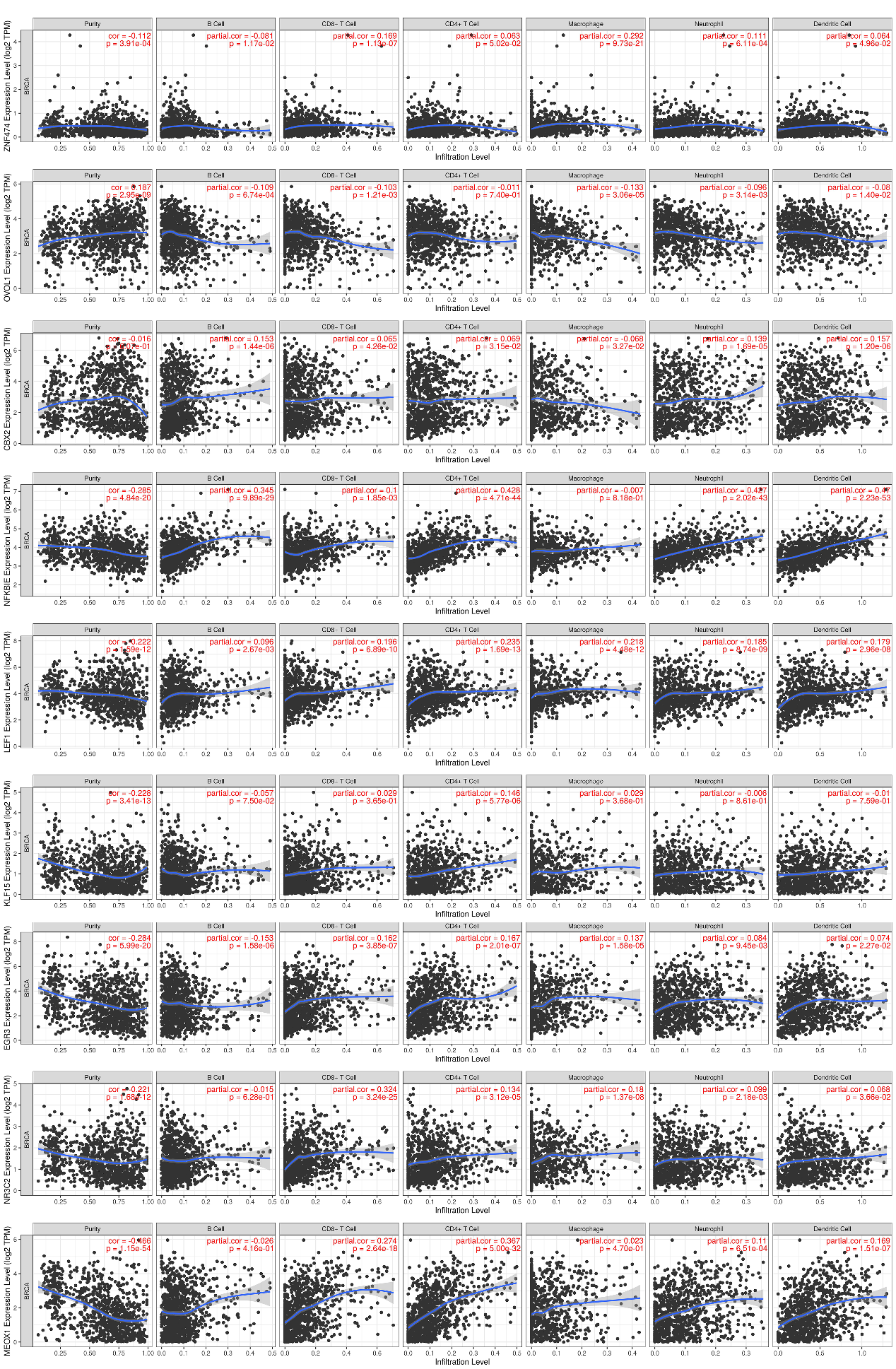


**Supplementary Figure S4.** **Correlations between expressions of TFDEGs and immune infiltration profiles in BRCA.** The figure shows the expression of each gene associated with tumor purity and several tumor-infiltrating immune cell markers, such as B cell, CD8+ T cell, CD4 + T cell, macrophage, neutrophil, and dendritic cell markers (*P* <0.05)
